# Biomimetic Macrophage Cell Membrane‐Based Nanoparticles for Effective Treatment of Glioblastoma Through Boron Neutron Capture Therapy Combined With Immunotherapy

**DOI:** 10.1002/advs.76216

**Published:** 2026-06-22

**Authors:** Jiawen Chen, Haoyu You, Hefa Huang, Huihui Chai, Ruize Zhu, Yun Guan, Qisheng Tang, Tianwen Li, Shan Jiang, Houshi Xu, Peng Wang, Yue Wang, Maoyuan Sun, Beining Liu, Zhen Li, Yulai Zeng, Weiqiu Ping, Yanlin Teng, Songlin Yan, Qinghui Li, Long Gu, Tao Sun, Zhifeng Shi

**Affiliations:** ^1^ Department of Neurosurgery Huashan Hospital Fudan University Shanghai China; ^2^ Neurosurgical Institute Fudan University Shanghai China; ^3^ Shanghai Clinical Medical Center of Neurosurgery Shanghai Key Laboratory of Brain Function and Restoration and Neural Regeneration Shanghai China; ^4^ Department of Pharmaceutics School of Pharmaceutical Sciences Key Laboratory of Smart Drug Delivery Ministry of Education State Key Laboratory of Brain Function and Disorders and MOE Frontiers Center For Brain Science Fudan University Shanghai China; ^5^ School of Nuclear Science and Technology Lanzhou University Lanzhou China; ^6^ Shanghai ImmunoBiotech Co., Ltd. Shanghai China

**Keywords:** biomimetic nanoparticles, blood‐brain barrier, boron neutron capture therapy, glioblastoma, immunotherapy

## Abstract

Glioblastoma multiforme (GBM) remains largely incurable due to the blood‐brain barrier (BBB) and immunosuppressive microenvironment. While boron neutron capture therapy (BNCT) selectively eradicates tumor cells via ^10^B(n, α)‐^7^Li reactions, its clinical potential in GBM is unrealized because of the suboptimal pharmacokinetics of conventional boron agents and their failure to achieve therapeutic intracranial concentrations. Here, we design macrophage membrane‐coated PLGA nanoparticles (M@PLGA‐^10^BN) that exploit innate biological transport mechanisms to overcome dual therapeutic barriers. The macrophage membrane enables: (i) α_4_β_1_ integrin‐mediated BBB transmigration via VCAM‐1 binding, (ii) chemokine‐directed tumor tropism, and (iii) CD47‐dependent immune evasion. In orthotopic GL261 gliomas, M@PLGA‐^10^BN delivers 53.15 µg/g ^10^B to tumors – 3.09‐fold higher than uncoated nanoparticles – with tumor‐to‐normal/brain (T/N) and tumor‐to‐blood (T/B) ratios of 3.95 and 3.85, respectively. Neutron irradiation extends survival by 50 days and triggers immunogenic cell death, increasing tumor‐infiltrating CD8^+^ T cells 4.2‐fold while reducing regulatory T cells (Tregs) by 68%. Crucially, BNCT‐induced PD‐L1 upregulation provides a mechanistic basis for combining with anti‐PD‐1 immunotherapy, achieving complete tumor regression in 50% of mice. By concurrently overcoming the blood‐brain barrier and immunosuppression, this biomimetic strategy unlocks new clinical horizons for BNCT, establishing a new paradigm for treating CNS malignancies.

## 1 Introduction

Glioblastoma (WHO Grade IV) portends a median survival under 15 months despite aggressive therapy [1]. Its resilience arises from three interconnected challenges: (1) The intact blood‐brain barrier (BBB) excludes >98% of systemically administered therapeutics, including structurally optimized agents; (2) Highly infiltrative growth defies surgical resection; (3) An immunosuppressive tumor microenvironment (TME), characterized by M2 macrophages and regulatory T cells (Tregs), subverts antitumor immunity [2, 3, 4].

Boron neutron capture therapy (BNCT) offers a compelling solution through binary precision: Tumor‐localized ^10^B captures thermal neutrons to generate high‐linear‐energy‐transfer α particles (1.47 MeV) and ^7^Li nuclei (0.84 MeV), confining lethal DNA damage within only 10 µm while sparing adjacent tissue. Although clinically approved for recurrent head/neck cancers, BNCT fails in GBM due to inadequate boron delivery. Current agents (BPA/BSH) achieve subtherapeutic tumor concentrations (<20 µg/g) and low T/N ratios (<2.5), exacerbated by BBB exclusion [5, 6, 7, 8].

Nanocarriers promise enhanced delivery but face critical limitations: Inorganic boron nanoparticles (e.g., BN, BPO_4_) incur systemic toxicity via reticuloendothelial uptake, while polymeric systems like PLGA suffer rapid opsonization and poor BBB penetration [9, 10, 11]. To address this, we pioneer a biomimetic strategy leveraging macrophage membranes‐nature's optimized biological transporters. Macrophage membrane‐coated nanoparticles retain functional α_4_β_1_ integrins that bind VCAM‐1 on inflamed BBB endothelium, enabling receptor‐mediated transcytosis. Simultaneously, chemokine receptors (e.g., CCR2) direct tumor homing, while surface CD47 confers “self‐signal” to evade phagocytosis [12, 13, 14, 15].

**SCHEME 1:**
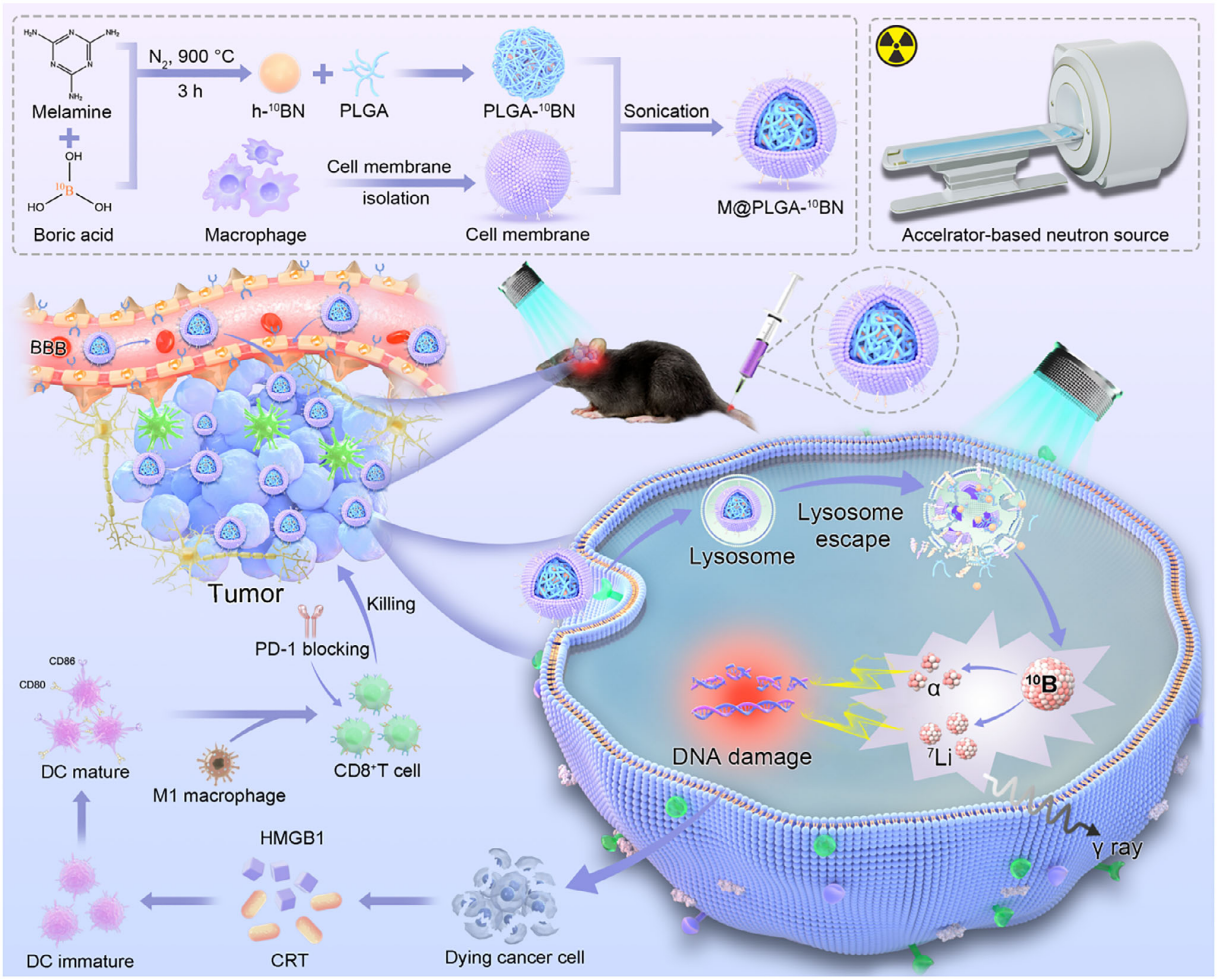
Schematic illustration of the synthesis of M@PLGA‐^10^BN NPs and its application for boron neutron capture therapy in an orthotopic glioblastoma mouse model.

We engineered M@PLGA‐^10^BN nanoparticles encapsulating biodegradable boron nitride (Scheme 1). This nanocomposite achieves triple functionality: (i) Prolonged circulation (8.5× longer than PEG‐PLGA) [11, 16], (ii) BBB penetration with 4.3× greater brain boron accumulation than BPA [17], and (iii) Deep tumor diffusion via size‐optimized chemotaxis. In orthotopic GBM models, it delivers 53.15 µg/g ^10^B to tumors–surpassing the therapeutic threshold (20 µg/g) – while maintaining favorable T/N and T/B ratios (3.95/3.85). Beyond direct tumor cytotoxicity, we discover BNCT remodels the immunosuppressive TME by inducing immunogenic cell death (calreticulin exposure, HMGB1 translocation) and recruiting cytotoxic CD8^+^ T cells. The consequent PD‐L1 upregulation reveals an adaptive resistance mechanism, informing rational combination with PD‐1 blockade that achieves curative outcomes. This work transforms GBM treatment by integrating targeted radiotherapy with immunotherapy through engineered biology.

## 2 Results

### 2.1 Preparation and Characterizations of the Nanocomposite

**FIGURE 1:**
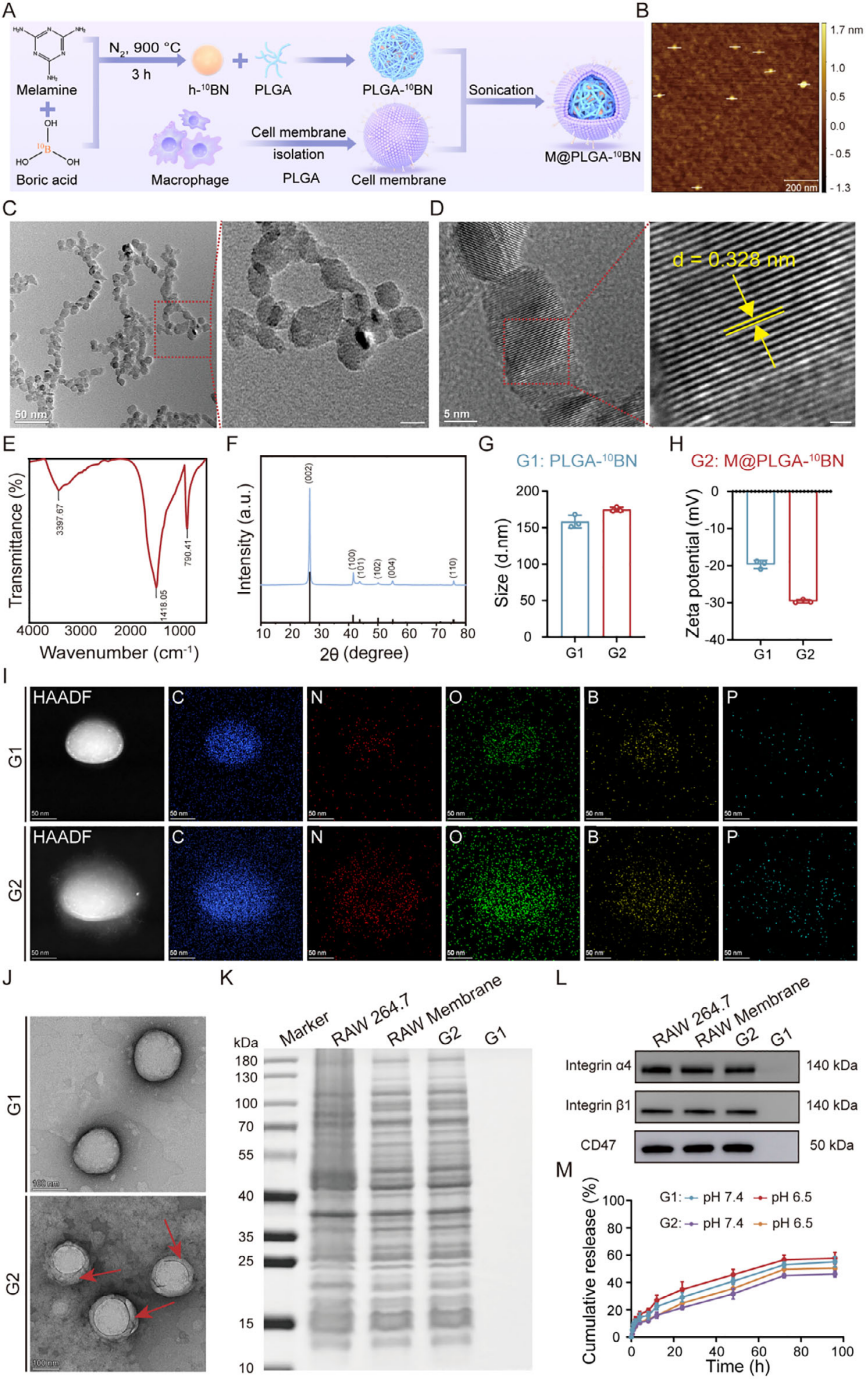
Preparation and characterizations of the nanocomposite. (A) Schematic illustration of the process used in the preparation of h‐^10^BN and M@PLGA‐^10^BN. (B) AFM image of h‐^10^BN nanoparticles. Scale bar = 200 nm. (C) TEM images of h‐^10^BN nanoparticles. Scale bar = 50 nm, Scale bar = 10 nm. (D) HRTEM image of h‐^10^BN nanoparticles. Scale bar = 5 nm, Scale bar = 1 nm. (E) FTIR spectra of h‐^10^BN nanoparticle. (F) Powder XRD scattering profile of h‐^10^BN nanoparticles. (G and H) Hydrodynamic particle size and zeta potential of PLGA‐^10^BN and M@PLGA‐^10^BN. (I) HAADF‐SETM images and element mapping of PLGA‐^10^BN and M@PLGA‐^10^BN. Scale bar = 50 nm. (J) Representative TEM images of PLGA‐^10^BN and M@PLGA‐^10^BN negatively stained with phosphotungstic acid. Scale bar = 100 nm. (K) SDS‐PAGE images of the protein profiles of macrophage cells (RAW 264.7), macrophage membrane, M@PLGA‐^10^BN. (L) Western blot images of proteins, including integrin α4 (140 kDa), integrin β1 (140 kDa), and CD47 (50 kDa), on the macrophage cells (RAW 264.7), macrophage membrane, PLGA‐^10^BN, and M@PLGA‐^10^BN. (M) In vitro release of ^10^BN from PLGA‐^10^BN and M@PLGA‐^10^BN in PBS (pH 7.4 and 6.5) at 37°C for 72 h with gentle stirring, respectively (n = 3 samples per group). Data were presented as mean ± SD.

The preparation procedures for 10‐boron nitride nanoparticles and M@PLGA‐^10^BN are illustrated in Figure 1A. First, ^10^B‐enriched boron nitride was synthesized through a bottom‐up pyrolytic method according to a previous study, using ^10^B‐boric acid (^10^B abundance: 99.43%) and melamine as ^10^B and nitrogen sources, respectively [18, 19, 20]. Transmission electron microscopy (TEM) revealed that the h‐^10^BN nanoparticles had an average diameter of approximately 8 nm; however, owing to their high surface energy, large aggregates were formed on the TEM grid (Figure 1C). High‐resolution transmission electron microscopy (HRTEM) analysis of the h‐^10^BN NPs revealed ordered fringes with a spacing of 0.328 nm, which were ascribed to the (002) and (100) facets of h‐^10^BN with layered features, confirming their high crystallinity (Figure 1D). The height distribution of the h‐^10^BN NPs was examined by atomic force microscopy (AFM), which revealed a height ranging from 2 to 5 nm (Figure 1B; Figure S1), which was consistent with the TEM and HRTEM observations. X‐ray photoelectron spectroscopy (XPS) analysis demonstrated that the h‐^10^BN NPs primarily consisted of four elements: boron, nitrogen, carbon, and oxygen, with a calculated B:N atomic ratio of 1.21:1.00 (Figure S2A). The B1s peak could be further deconvoluted into two characteristic peaks at 190.02 and 191.16 eV, corresponding to B─N and B─O bonds, respectively (Figure S2B). Similarly, the N1s peak exhibited two characteristic peaks at 398.13 and 398.84 eV, which are attributed to N‐B and N─H bonds, respectively (Figure S2C). Fourier transform infrared spectroscopy (FTIR) of h‐^10^BN NPs results showed a broad absorption band in the range of 3000–3700 cm^−^^1^, attributable to the stretching vibrations of O─H and N─H bonds (Figure 1E). Further analysis confirmed the existence of abundant boric acid groups (‐B(OH)_2_) and amino groups (‐NH_2_) on the NP surface, and was probably generated on the edges of (002) plane during the water‐etching process. FTIR exhibited two strong peaks at 1418.05 and 790 cm^−^^1^, which corresponded to the stretching and bending vibrations of ^10^B‐N, respectively (Figure 1E). X‐ray diffraction (XRD) was used to investigate the crystal structure and phase composition of ^10^BN NPs. As shown in Figure 1F, the h‐^10^BN nanoparticles exhibit excellent crystallinity, with diffraction peaks at 26.7°, 41.6°, and 43.8°, corresponding to the (002), (100), and (101) crystal facets of h‐^10^BN, respectively. To evaluate the surface charge and stability of h‐^10^BN NPs, their particle size and zeta potential were measured. The results of dynamic light scattering (DLS) indicated that owing to aggregation, h‐^10^BN NPs exhibited a median hydrodynamic diameter of up to 18.36 nm in water (Figure S3). Owing to the acidic nature of the ‐B(OH)_2_ groups on the nanoparticle surface, the zeta potential of h‐^10^BN NPs was measured as −30.43 mV (Figure S4).

Although h‐^10^BN NPs can initially be dispersed in pure water because of their high surface negative charge and abundant hydrophilic groups, they tend to aggregate and precipitate in physiological solutions such as phosphate‐buffered saline (PBS) because of the electron‐shielding effect [18, 19, 21]. Previous studies have employed polyglycerol (PG) and polyethylene glycol (PEG) modifications of boron nitride NPs to enhance their dispersibility and colloidal stability under physiological conditions, thereby facilitating biomedical applications [18]. However, these methods have drawbacks such as complex procedures and stringent experimental requirements. Therefore, we explored the encapsulation of ^10^BN nanoparticles with PLGA polymer nanomaterials, followed by macrophage coating to prepare ^10^BN‐based biomimetic nanoparticles. Based on previous studies, the emulsion‐solvent evaporation method was successfully employed to fabricate PLGA‐^10^BN. DLS analysis revealed that PLGA‐^10^BN exhibited an average hydrodynamic diameter of approximately 158.2 nm (Figure 1G) and an average zeta potential of −19.6 mV (Figure 1H). Macrophages were subsequently collected, and purified macrophage membranes were obtained through hypotonic swelling, followed by physical homogenization. Next, the purified macrophage membranes were coated onto PLGA‐^10^BN via ultrasonic processing to prepare M@PLGA‐^10^BN, which exhibited an average hydrodynamic diameter of approximately 174.7 nm (Figure 1G) and a mean zeta potential of −29.6 mV (Figure 1H). Compared with PLGA‐^10^BN, the increased average size and surface charge of M@PLGA‐^10^BN confirmed the successful coating of the macrophage membranes on the PLGA‐^10^BN surface. Furthermore, high‐angle annular dark‐field scanning transmission electron microscopy (HAADF‐STEM) and elemental mapping of both types of NPs confirmed the even distribution of B and N within the NPs, demonstrating the effective PLGA encapsulation of boron nitride NPs (Figure 1I). We also observed a uniform distribution of P within the M@PLGA‐^10^BN NPs compared to PLGA‐^10^BN (Figure 1I), indicating the successful coating of the macrophage membrane onto PLGA‐^10^BN. Transmission electron microscope (TEM) was employed to observe the morphology of the two nanoparticles and to further verify whether the macrophage membranes were successfully coated on the PLGA‐^10^BN surface. Compared to the naked spherical PLGA cores, the TEM images revealed the typical sphere‐like core–shell structure of M@PLGA‐^10^BN (Figure 1J). Further particle size measurements showed that PLGA‐^10^BN had an average size of 144.2 nm, while that of M@PLGA‐^10^BN was 162.1 nm (Figure 1J). Subsequently, the protein composition of the macrophage membranes in the M@PLGA‐^10^BN was analyzed. SDS‐PAGE demonstrated that the protein profile of the M@PLGA‐^10^BN NPs closely resembled that of macrophage lysates (containing intracellular proteins; Figure 1K), with some protein bands observed at approximately 180, 130, 100, 70, 50, 37, and 25 kDa, which indicated successful retention of the macrophage membrane architecture on the nanoparticles. Moreover, crucial proteins responsible for BBB penetration and immune evasion, including integrin α4 (140 kDa), integrin β1 (140 kDa), and CD47 (50 kDa), were all observed in macrophage lysates, macrophage membrane and M@PLGA‐^10^BN group (Figure 1L). These results collectively confirm the successful encapsulation of macrophage membranes onto the PLGA‐^10^BN core surface with preserved targeting capability.

Finally, the size stability and the release behaviors of the two NPs were evaluated in vitro. NP size variations were continuously monitored in PBS. Compared to PLGA‐^10^BN, M@PLGA‐^10^BN exhibited superior colloidal stability, indicating a protective effect conferred by the macrophage membrane coating (Figure S5). Furthermore, this enhanced stability suggests that M@PLGA‐^10^BN may possess improved biocompatibility and a prolonged circulation time in vivo, thereby achieving more effective therapeutic outcomes. Notably, the release rate of ^10^BN from M@PLGA‐^10^BN was lower than that from uncoated PLGA‐^10^BN nanoparticles at both pH 7.4 and 6.5, suggesting that the macrophage membrane functions as an effective diffusion barrier that retards drug release. Additionally, a mildly acidic condition (pH 6.5) facilitated slightly greater ^10^BN release from the nanoparticles relative to the neutral condition (pH 7.4) (Figure 1M). Collectively, these results demonstrated that M@PLGA‐^10^BN exhibited favorable stability and sustained‐release properties in vitro.

### 2.2 Cellular Uptake and BBB Penetration of the Nanocomposite In Vitro

**FIGURE 2:**
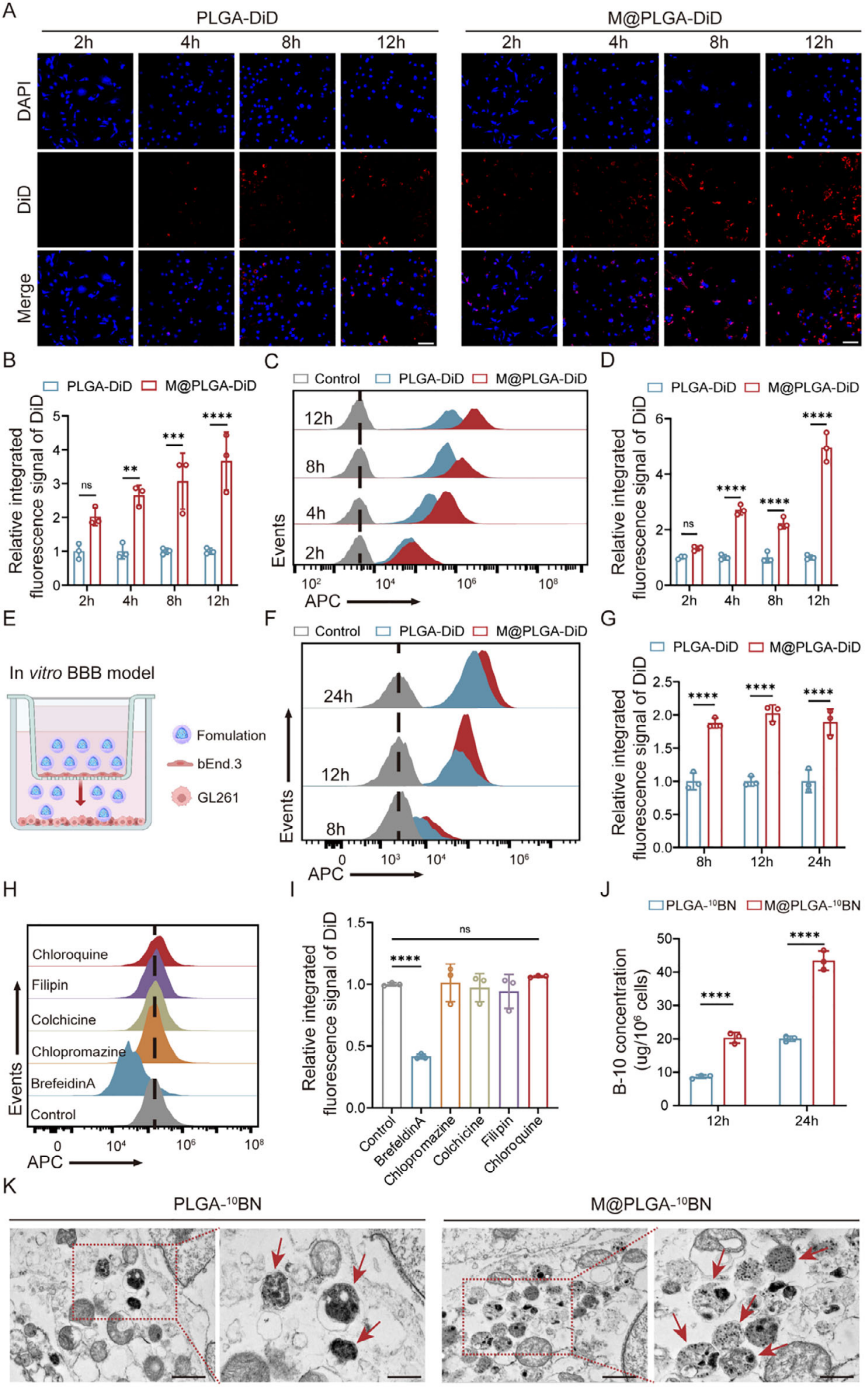
The cellular uptake and BBB crossing by nanocomposite in vitro. (A and B) Representative CLSM images showed the cell internalizations of PLGA‐DiD and M@PLGA‐DiD into GL261 cells at various timepoints along with the corresponding results from the semi‐quantitative analysis. Scale bar = 100 µm. (C and D) Flow cytometry‐based analysis of the uptake of PBS, PLGA‐DiD and M@PLGA‐DiD incubated with GL261 cells for various timepoints and corresponding results from the semi‐quantitative analysis. (E) Schematic diagram of the in vitro BBB model transwell assay with GL261 cells seeded on the bottom chamber. (F and G) Flow cytometry‐based analysis showed the cell internalization of PBS, PLGA‐DiD and M@PLGA‐DiD into GL261 cells at various timepoints and the corresponding results from the semi‐quantitative analysis. (H and I) Flow cytometry‐based analysis of the uptake pathway of M@PLGA‐DiD and the corresponding results from the semi‐quantitative analysis. (J) The boron concentrations of GL261 cells incubated with PLGA‐^10^BN and M@PLGA‐^10^BN (^10^B concentration: 100 µg/mL) in each period. (K) Representative TEM images showed the cell internalization of PLGA‐^10^BN and M@PLGA‐^10^BN into GL261 cells at 24 h in the in vitro BBB model. Scale bar = 1 µm, Scale bar = 500 nm. Statistical significance was calculated via one‐way ANOVA with Tukey's test: ns: non‐significant, ^*^ *p*<0.05, ^**^ *p*<0.01, ^***^ *p*<0.001, ^****^ *p*<0.0001.

To evaluate the cellular uptake of nanoparticles by tumor cells in vitro, we fabricated two types of NPs—PLGA‐DiD and M@PLGA‐DiD. Initially, both NPs were co‐incubated with GL261 cells ex vivo at identical concentrations for various durations. Confocal microscopy revealed that M@PLGA‐DiD exhibited superior cellular uptake by GL261 cells compared to PLGA‐DiD, with the uptake increasing over time, which indicated time‐dependent internalization (Figure 2A). Quantitative fluorescence analysis demonstrated that after 12 h of co‐incubation, the cellular uptake of M@PLGA‐DiD was approximately 3.5‐fold higher than that of PLGA‐DiD (Figure 2B). Flow cytometry further corroborated these findings, showing consistent results in nanoparticle uptake (Figure 2C,D). Collectively, these data indicate that the macrophage membrane coating significantly enhances the uptake of NPs by tumor cells.

The integrin α_4_β_1_ on macrophage membranes can bind to vascular cell adhesion molecule‐1 (VCAM‐1) on the BBB, and thereby mediate the trans‐BBB migration and tumor tissue accumulation of NPs [12, 14]. To evaluate the BBB‐penetrating capabilities of the two NPs, we established an ex vivo BBB model using a previously described method [13]. bEnd.3 cells were seeded in the upper chamber of the transwell inserts at a density of 2 × 10^4^ cells/well. The upper chamber was filled with 200 µL complete medium whereas the lower chamber contained 800 µL of complete medium. The medium in both chambers was replaced every 2 days until day 14. Transendothelial electrical resistance (TEER) was measured every 48 h. When TEER values exceeded 300 Ω·cm^2^, tumor cells were cultured in the lower chamber for 24 h. Different formulations were added to the upper chambers for different incubation periods (Figure 2E). The results demonstrated that M@PLGA‐DiD effectively crossed the BBB and was efficiently internalized by the glioma cells (Figure 2F). Compared to the PLGA‐DiD group, the fluorescence intensity of M@PLGA‐DiD in tumor cells increased approximately 2‐fold (Figure 2G). Furthermore, intracellular NP accumulation exhibited a distinct time‐dependent enhancement (Figure 2F,G). Given that ^10^BN primarily exerts intracellular effects, we investigated its uptake mechanism at the cellular level. Studies revealed that GL261 cells primarily internalize M@PLGA‐DiD through clathrin‐mediated endocytosis (Figure 2H,I). To further investigate whether the biomimetic nanoparticles possess lysosomal escape capability upon entering lysosomes, we co‐incubated the M@PLGA‐DiD with cells and visualized their intracellular localization with respect to lysosomes using immunofluorescence. As shown in Figure S6, at 2 h, the DiD red fluorescence signal representing the M@PLGA‐DiD was mainly localized on the cell membrane, suggesting that the majority of M@PLGA‐DiD had not yet been fully internalized. At 4 h, the red signal began to co‐localize with the green signal (LysoTracker), confirming the presence of the M@PLGA‐DiD within lysosomes. Notably, at 8 h, the M@PLGA‐DiD exhibited markedly reduced lysosomal co‐localization, as evidenced by diminished green fluorescence and extensive red fluorescence dispersal into the cytoplasm. These observations collectively indicate that the nanocomposite successfully escapes from lysosomes, confirming its lysosomal escape capability.

Finally, the ex vivo biological characteristics of PLGA‐^10^BN and M@PLGA‐^10^BN were investigated. The cytotoxic effects of PLGA‐^10^BN and M@PLGA‐^10^BN were evaluated. The NPs were co‐cultured with GL261 cells in vitro for 12, 24, 48, and 72 h, followed by assessment of tumor cell viability. The CCK‐8 assay results indicated no significant cytotoxicity among the different concentration groups at 12, 24, 48 or 72 h (Figure S7). Based on the CCK‐8 experimental results and previous studies, a ^10^B concentration of 100 µg/mL was selected for subsequent in vitro experiments [22, 23]. BNCT‐induced tumor cell killing requires efficient cellular uptake; thus, high uptake of both types of NPs by tumor cells is a prerequisite for BNCT. GL261 cells were co‐incubated with the two nanoparticles for 12 and 24 h, washed three times with PBS, and the boron content in 1 × 10^6^ cells was determined using inductively coupled plasma mass spectrometry (ICP‐MS). ICP‐MS analysis revealed that after 12 and 24 h of co‐incubation with PLGA‐^10^BN, the average boron content per 10^6^ cells was 8.62 and 20.00 µg, respectively. In contrast, the M@PLGA‐^10^BN‐treated group exhibited significantly higher boron accumulation, reaching 20.28 and 43.43 µg, respectively (Figure 2J). These results demonstrate that both nanoparticles enabled intracellular boron accumulation to meet the requirements for BNCT after 24‐h incubation, providing experimental evidence for the feasibility of BNCT for in vitro tumor‐cell killing. Subsequently, we sought to verify whether PLGA‐^10^BN and M@PLGA‐^10^BN could cross the BBB in vitro and be internalized by tumor cells. Similarly, an ex vivo BBB model was established, and tumor cells were collected after 24 h for TEM observation. The TEM results revealed the presence of NPs within the tumor cells. NPs were primarily localized in the cytoplasm, as indicated by red arrows (Figure 2K). Moreover, the quantity of M@PLGA‐^10^BN in tumor cells was significantly higher than that of PLGA‐^10^BN (Figure 2K). These findings demonstrate that M@PLGA‐^10^BN exhibits superior BBB penetration and tumor cell uptake compared with PLGA‐^10^BN.

### 2.3 Tumor Targeting and Biodistribution of the Nanocomposite

**FIGURE 3:**
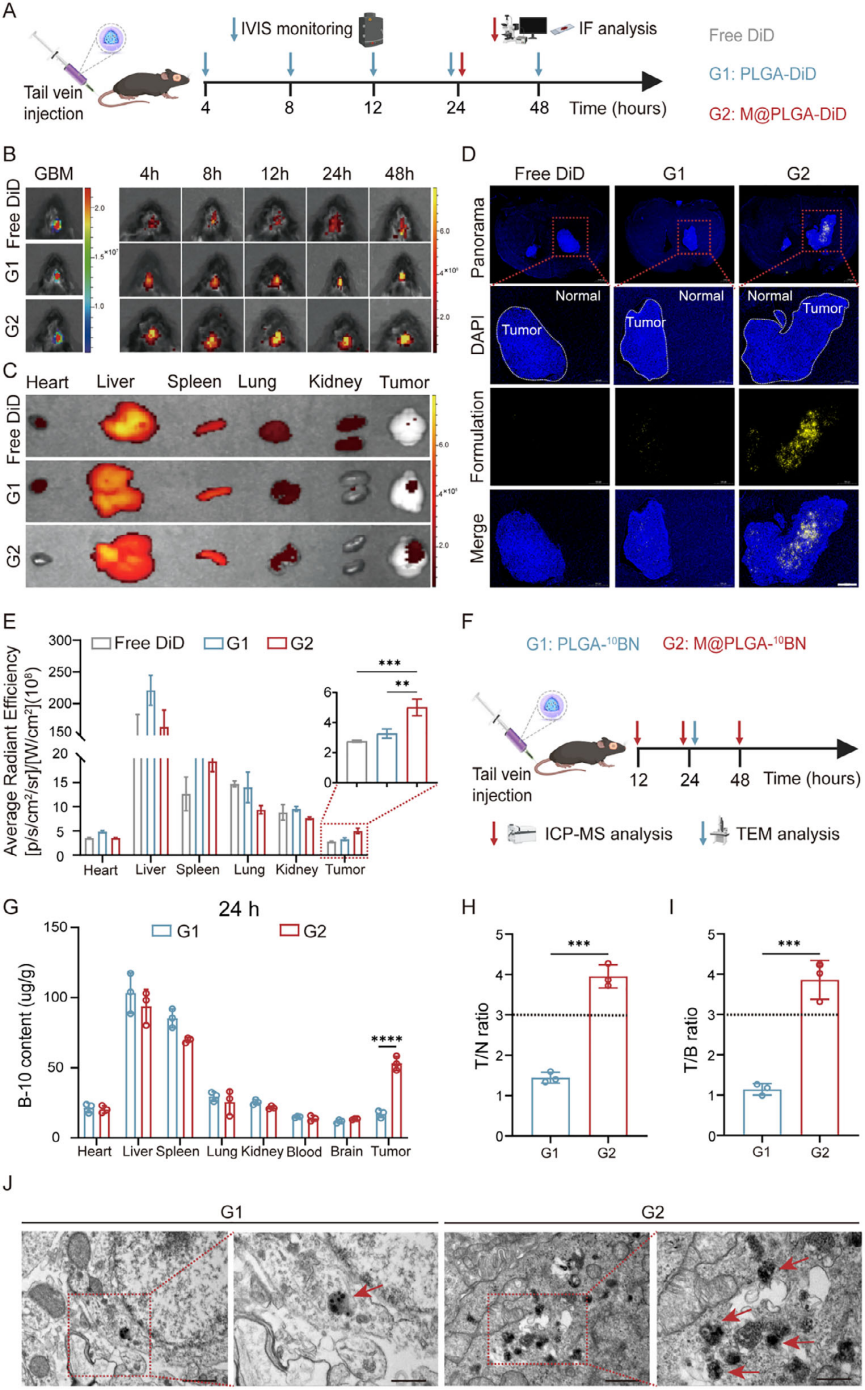
Tumor targeting and biodistribution of the nanocomposite. (A) Schedule of the mouse experiments for tumor targeting and biodistribution analysis in the administration groups. (B) Real‐time bioluminescent fluorescence images of mice bearing orthotopic glioma tumor at various timepoints after i.v. injection of free DiD, PLGA‐DiD, and M@PLGA‐DiD. (C) Ex vivo bioluminescent fluorescence images of brains and tissues (heart, liver, spleen, lung, and kidney) harvested from mice bearing orthotopic glioma tumor at 24 h after i.v. injection of free DiD, PLGA‐DiD, and M@PLGA‐DiD. (D) Representative fluorescence microscopy images of DAPI (blue), and DiD (yellow) staining in brains bearing GBM at 24 h after i.v. injection of free DiD, PLGA‐DiD, and M@PLGA‐DiD. Scale bar = 1000 µm, Scale bar = 500 µm. (E) Semi‐quantitative analysis of the fluorescence intensity of the brains and tissues at 24 h after i.v. injection of free DiD, PLGA‐DiD, and M@PLGA‐DiD. Data presented are the mean ± SD (n = 3). (F) Schedule of the mouse experiments for boron concentration of the brains and tissues in different groups. (G) The boron concentration of the brains and tissues at 24 h after i.v. injection of PLGA‐^10^BN and M@PLGA‐^10^BN. Data presented are the mean ± SD (n = 3). (H and I) The ratio of boron levels in tumor‐to‐blood (T/B) and the ratio of boron levels in tumor‐to‐normal tissue (T/N) at 24 h after i.v. injection of PLGA‐^10^BN and M@PLGA‐^10^BN. Data presented are the mean ± SD (n = 3). (J) Representative TEM images showing the cellular infiltration and decomposition of PLGA‐^10^BN and M@PLGA‐^10^BN in glioma tumor tissues. Scale bar = 1 µm, Scale bar = 500 nm. Statistical significance was calculated via one‐way ANOVA with Tukey's test: ns: non‐significant, ^*^ *p*<0.05, ^**^ *p*<0.01, ^***^ *p*<0.001, ^****^ *p*<0.0001.

As previously demonstrated, M@PLGA‐^10^BN exhibited excellent BBB penetration and tumor cell uptake properties in vitro. Subsequently, we evaluated the in vivo tumor‐targeting capability and biodistribution of both NPs (Figure 3A). Since in vivo fluorescence imaging enables real‐time monitoring of nanoparticle tumor targeting and biodistribution, we prepared PLGA‐DiD and M@PLGA‐DiD as described earlier. First, we assessed the in vivo brain targeting efficiency of M@PLGA‐DiD. GL261 glioma cells expressing luciferase were orthotopically implanted into the brains of C57BL/6 mice, and tumor growth was confirmed using an in vivo imaging system (IVIS). Subsequently, equal concentrations of the nanoparticle formulations were administered via tail vein injection. The results revealed that significant NP accumulation in the tumor region was observed as early as 4 h post injection with M@PLGA‐DiD, as evidenced by the strong signal from the DiD fluorescent probe. Notably, NP accumulation peaked at 24 h, followed by a gradual decline in the signal, although detectable fluorescence persisted for up to 48 h (Figure 3B). To investigate nonspecific distribution, mice were sacrificed at 24 h post‐injection, and major organs were harvested for ex vivo fluorescence imaging. As shown in Figure 3C,E, M@PLGA‐DiD NPs generally exhibited lower fluorescence signals in the heart, liver, and spleen than PLGA‐DiD, whereas strong fluorescence signals in glioma tissue confirmed the significant tumor accumulation of M@PLGA‐DiD. These findings indicate that M@PLGA‐DiD possess superior brain‐targeting capabilities. This effect may be attributed to two factors: (1) the macrophage membrane itself enhances the brain‐targeting ability of the nanocomposite, and (2) the macrophage membrane coating on the nanoparticle surface effectively constructs a protective layer, thereby reducing the likelihood of hepatic metabolic clearance and phagocytic capture by the spleen. Thus, macrophage membrane modification not only improves the brain‐targeting performance of the nanocomposite, but also minimizes its accumulation in the liver and spleen, effectively avoiding non‐specific distribution. Notably, in brain sections obtained 24 h after tail‐vein injection, significantly higher accumulation of M@PLGA‐DiD was observed in glioma tissue lesions than in PLGA‐DiD (Figure 3D). These results indicate that M@PLGA‐DiD effectively penetrate the BBB to reach the glioma tissue, whereas PLGA‐DiD without macrophage membrane modification remain “trapped” within blood vessels, failing to cross the BBB. Collectively, these experimental results demonstrate that M@PLGA‐DiD serves as an excellent brain‐delivery platform with superior BBB‐penetrating capabilities and specific glioma‐targeting properties.

For BNCT efficacy, tumor tissue must achieve ≥20 µg/g ^10^B concentration with T/N and T/B ratios ≥3 [24]. To evaluate the biodistribution characteristics of nanoparticles in glioma‐bearing mice, we quantified the boron content in various tissues using ICP‐MS following the administration of equivalent doses (Figure 3F). After intravenous injection of NPs (^10^B, 50 mg/kg) via the tail vein, the glioma‐bearing mice were sacrificed at 12, 24, and 48 h post‐injection. The heart, liver, spleen, lung, kidney, blood, tumor tissue, and normal brain tissue were collected for ICP‐MS analysis. The results demonstrated time‐dependent changes in ^10^B concentrations across tissues, peaking at 24 h before gradually declining (Figure 3G; Figure S8). Notably, the M@PLGA‐^10^BN group exhibited significantly higher ^10^B accumulation in tumor tissue (53.15 µg/g) compared to the PLGA‐^10^BN group (17.16 µg/g), which exceeded the minimum therapeutic threshold for BNCT (Figure 3G). Furthermore, the T/N and T/B ratios in the M@PLGA‐^10^BN group were 3.95 and 3.85, respectively, which were significantly higher than those in the PLGA‐^10^BN group (Figure 3H,I). Collectively, these data confirmed that M@PLGA‐^10^BN met the boron delivery requirements for effective BNCT.

TEM was used to examine intracellular nanoparticle distribution. TEM images revealed substantially more NPs in the cytoplasm of tumor cells from the M@PLGA‐^10^BN group than from the PLGA‐^10^BN group (Figure 3J), which demonstrated that the NPs could be endocytosed and reside in the cells of tumor tissues. Interestingly, the spherical morphology of some M@PLGA‐^10^BN NPs changed to an irregular structure, which indicated that some NPs were degraded inside the cells.

### 2.4 BNCT Efficacy Evaluation In Vitro

**FIGURE 4:**
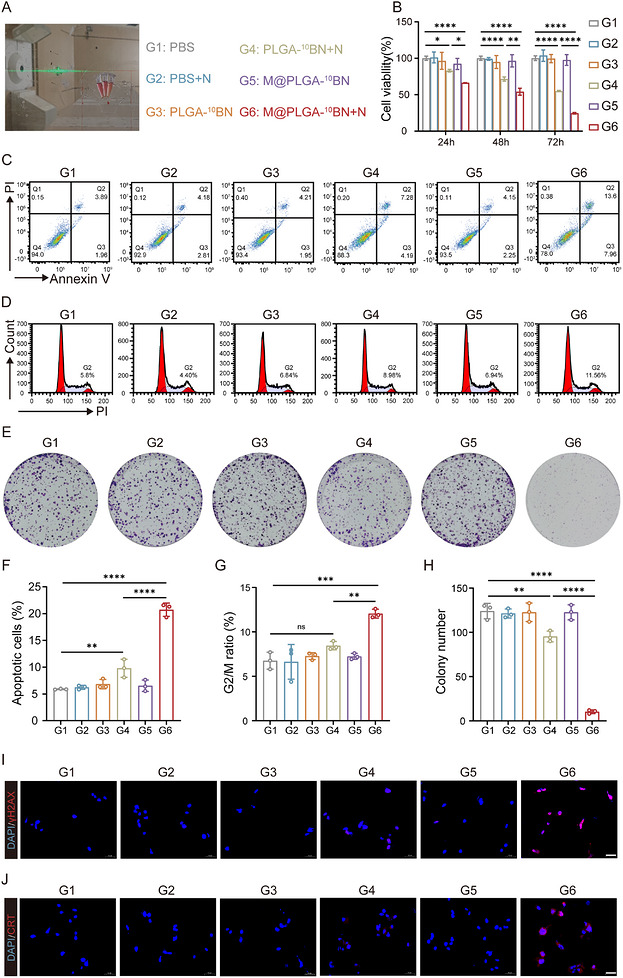
The BNCT efficacy evaluation in vitro. (A) The cell position when accepted with neutron irradiation. (B) Cell viability of GL261 cells was assessed on the second day after neutron irradiation in different groups. (C) Flow cytometry‐based analysis of apoptosis in GL261 cells on the second day after neutron irradiation in different groups. (D) Flow cytometry‐based analysis of the cell cycle in GL261 cells on the second day after neutron irradiation in different groups. (E) Colony‐formation assay of GL261 cells cultured in six‐well plates after neutron irradiation in different groups. (F) The results of the quantitative analysis of apoptosis in GL261 cells in C (data represent mean ± SD, n = 3). (G) The results of the quantitative analysis of the cell cycle in GL261 cells in D (data represent mean ± SD, n = 3). (H) The results of the quantitative analysis of colony formation in GL261 cells in E (data represent mean ± SD, n = 3). (I) Representative immunofluorescence images of γ‐H2AX in GL261 cells on the second day after neutron irradiation in different groups. Scale bar = 50 µm. (J) Representative immunofluorescence images of the expression of CRT in GL261 cells on the second day after neutron irradiation in different groups. Scale bar = 50 µm. Statistical significance was calculated via one‐way ANOVA with Tukey's test: ns: non‐significant, ^*^ *p*<0.05, ^**^ *p*<0.01, ^***^ *p*<0.001, ^****^ *p*<0.0001.

As previously described, the intracellular ^10^B content after 24‐h co‐culture with nanoparticles was sufficient to perform BNCT in vitro. We subsequently evaluated the antitumor efficacy of both types of nanoparticles in BNCT using in vitro experiments. GL261 cells were incubated with two types of NPs (^10^B, 100 µg/mL) for 24 h. The cells were then collected into EP tubes and subjected to neutron irradiation for 20 min (neutron flux: 6.96×10^8^ n cm^−2^ s^−1^). During irradiation, the cells were positioned on the device and exposed to neutron beams at identical doses (Figure 4A). For the in vitro cell viability assessment, irradiated cells were seeded in 96‐well plates at a density of 5 × 10^3^ cells/well and cultured for varying durations. The CCK‐8 assay results demonstrated a time‐dependent decrease in cell viability for both the PLGA‐^10^BN+N and M@PLGA‐^10^BN+N treatment groups, showing statistically significant differences compared to the controls (Figure 4B). Notably, the M@PLGA‐^10^BN+N group exhibited superior proliferation inhibition, with significantly lower viability at all timepoints compared with the PLGA‐^10^B+N group (Figure 4B). After 72‐h culture, the viability of the M@PLGA‐^10^BN+N group decreased to 24.50%, whereas the PLGA‐^10^BN group maintained 55.03%, further confirming enhanced antitumor effects of M@PLGA‐^10^BN (Figure 4B). BNCT induces apoptosis through mitochondrial pathways and causes G2/M phase cell‐cycle arrest [25]. To evaluate the cytotoxic effects of BNCT, we systematically analyzed the cellular apoptosis following neutron irradiation. Flow cytometry‐based analysis after 48‐h culture revealed significantly elevated apoptosis rates in both the PLGA‐^10^BN+N and M@PLGA‐^10^BN+N groups compared to the untreated controls (Figure 4C). Quantitative analysis demonstrated the M@PLGA‐^10^BN+N group achieved 20.73% apoptosis, showing statistical significance compared with PLGA‐^10^BN+N (12.15%) and control (4.32%) groups (Figure 4F). These results confirmed that M@PLGA‐^10^BN+N‐mediated BNCT effectively induced tumor cell apoptosis, representing a key antitumor mechanism. Next, we analyzed the effects of BNCT on cell‐cycle progression. Flow cytometry revealed that the proportion of cells in the G2/M phase was slightly higher in the PLGA‐^10^BN+N group (8.44%) that in the untreated control group (6.76%), although this difference was not statistically significant. In contrast, the M@PLGA‐^10^BN+N treatment group exhibited a significant increase in the G2/M phase population (12.05%), demonstrating statistically significant differences compared to both the control and PLGA‐^10^BN+N groups (Figure 4D,G). Clonal formation experiments were performed to assess the ability of the individual cells to grow into colonies. Following irradiation, the cells were seeded into culture dishes, fixed, and stained with crystal violet after 10 days of culture. The number of colonies formed was quantified. Compared with the other control groups, tumor cell proliferation was significantly suppressed in the M@PLGA‐^10^BN+N treatment group (Figure 4E,H). The survival fraction results from the clonogenic assay showed that the survival fractions of the PLGA‐^10^BN+N group and the M@PLGA‐^10^BN+N group were both significantly lower than that of the control group, with values of 76.88% and 8.33%, respectively. Moreover, the survival fraction of the M@PLGA‐^10^BN+N group was significantly lower than that of the PLGA‐^10^BN+N group (Figure S9). These results demonstrated that M@PLGA‐^10^BN effectively inhibited the growth of glioma cells following neutron irradiation. Collectively, these results indicate that M@PLGA‐^10^BN combined with neutron irradiation can effectively suppress glioma cell growth, as evidenced by the cell‐cycle arrest and reduced clonogenic capacity.

The antitumor efficacy of BNCT relies primarily on neutron irradiation‐induced double‐strand DNA breaks (DSBs), which subsequently induce apoptosis. As a sensitive marker of DSBs, the phosphorylation of histone H2AX at Ser139 (γ‐H2AX) rapidly responds to DNA damage [6, 8]. To evaluate the DNA damage effects of BNCT, γ‐H2AX foci formation was detected by immunofluorescence at 48 h post‐neutron irradiation. The results demonstrated that both the PLGA‐^10^BN+N group and M@PLGA‐^10^BN+N group exhibited significantly enhanced γ‐H2AX fluorescence intensity as compared with the untreated control group. Notably, the fluorescence intensity of the M@PLGA‐^10^BN + N group was higher than that of the PLGA‐^10^BN+N group (Figure 4I; Figure S10A). As per its working principle, DNA damage‐induced cell death as a primary therapeutic mechanism of BNCT. However, recent studies have suggested that BNCT may induce sustained antitumor efficacy by reprogramming the immunosuppressive tumor immune microenvironment (TIM) into an immunostimulatory state. Immune activation is triggered by therapy‐induced immunogenic cell death (ICD), which is characterized by the release of damage‐associated molecular patterns (DAMPs) such as calreticulin (CRT) and high‐mobility group box 1 (HMGB1) from dying tumor cells [9, 18, 26]. To investigate whether BNCT enhances anti‐glioma efficacy through ICD, we examined the expression of key DAMPs at 48 h post‐treatment. Immunofluorescence analysis and flow cytometry revealed that CRT exposure was markedly increased in both treatment groups, and the M@PLGA‐^10^BN+N group exhibited higher CRT levels than the PLGA‐^10^BN+N group (Figure 4J; Figure S10B–D). Concurrently, the subcellular localization analysis of HMGB1 demonstrated pronounced nuclear translocation in both treatment groups, with M@PLGA‐^10^BN+N exhibiting significantly greater translocation than PLGA‐^10^BN+N (Figure S10E–H).

Collectively, these findings indicate that BNCT treatment, particularly M@PLGA‐^10^BN mediated BNCT, effectively induces two critical ICD hallmark events—CRT exposure and HMGB1 nuclear translocation—which may contribute to sustained antitumor efficacy.

### 2.5 Efficacy of BNCT Evaluation In Vivo

**FIGURE 5:**
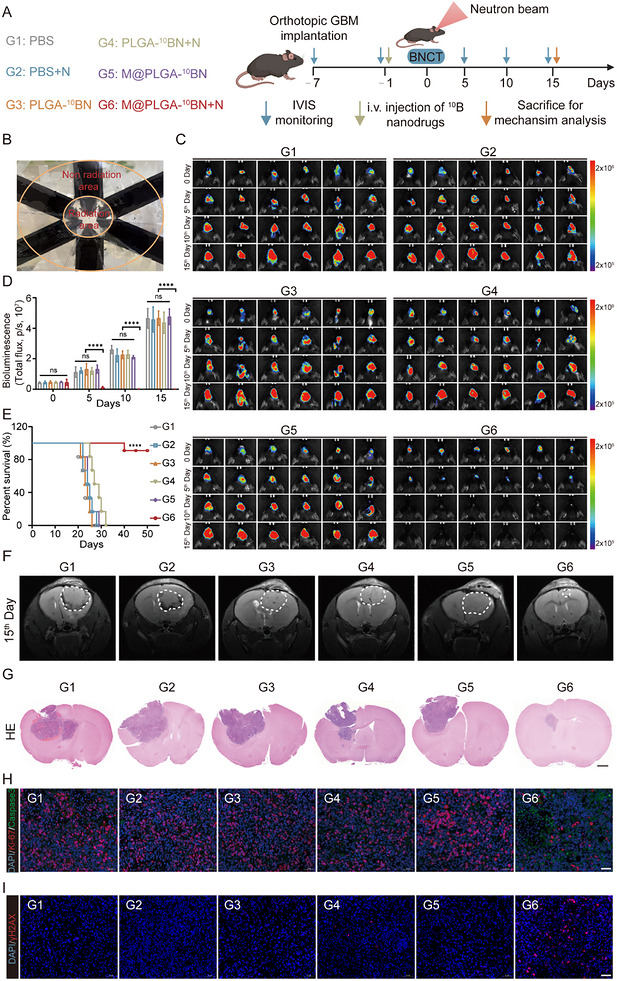
The efficacy of BNCT evaluation in vivo. (A) Schematic illustration of the administration groups and regimens of orthotopic GBM tumor‐bearing mice. (B) Illustration of the mouse‐fixation apparatus. The mouse brain was fixed within the neutron exposure area when administering BNCT. (C) Real‐time bioluminescent fluorescence images of mice bearing orthotopic GL261‐Luc glioma tumor at various timepoints after neutron irradiation in different groups. (D) Quantitative real‐time bioluminescent fluorescent analysis in mice bearing orthotopic GL261‐Luc glioma tumors. Data shown are the mean ± SD (n = 6). (E) Kaplan−Meier survival analysis of GL261‐Luc glioma tumor‐bearing mice after neutron irradiation in different groups. (F) Representative MRI images on the 15th day after neutron irradiation in different groups. (G) H&E staining images in tumor tissues from GL261‐Luc glioma tumor‐bearing mice in different groups. Scale bar = 1000 µm. (H) Representative immunofluorescence staining of Ki‐67 (red) and caspase‐3 (green) in tumors from different groups. Scale bar = 50 µm. (I) Representative immunofluorescence images of γ‐H2AX in tumor sections. Scale bar = 50 µm. Statistical significance was calculated via one‐way ANOVA with Tukey's test: ns: non‐significant, ^*^ *p*<0.05, ^**^ *p*<0.01, ^***^ *p*<0.001, ^****^ *p*<0.0001.

To evaluate the antitumor efficacy of NP‐mediated BNCT in vivo, an orthotopic xenograft model was established using GL261‐Luc cells. The experimental workflow for animal studies is shown in Figure 5A. On day 7, tumor‐bearing mice were randomly divided into six groups: PBS, PBS+N, PLGA‐^10^BN, PLGA‐^10^BN+N, M@PLGA‐^10^BN, and M@PLGA‐^10^BN+N (^10^B, 50 mg/kg body weight). Neutron irradiation was performed 24 h after intravenous administration (Figure 5B). IVIS bioluminescence imaging revealed that the tumor fluorescence signal in the M@PLGA‐^10^BN+N group exhibited progressive attenuation over time, whereas all the other groups, including the PLGA‐^10^BN+N group, demonstrated sustained enhancement (Figure 5C). Quantitative analysis indicated that the relative fluorescence intensity in the M@PLGA‐^10^BN+N group was significantly lower than those in the PBS and PLGA‐^10^BN+N groups after irradiation (Figure 5D). Although the PLGA‐^10^BN+N group showed reduced fluorescence signals compared with the PBS group, the difference was not statistically significant. This observation may be attributed to the insufficient ^10^B content (<20 µg/g) in tumor tissues (Figure 3G). The MRI results are consistent with the bioluminescence imaging data. M@PLGA‐^10^BN+N‐treated mice showed a significant reduction in the intracranial tumor volume, whereas the PLGA‐^10^BN+N and PBS control groups exhibited evident tumor progression (Figure 5F). These results further confirmed the remarkable antitumor effects of M@PLGA‐^10^BN‐mediated BNCT. The survival time of the tumor‐bearing mice treated with M@PLGA‐^10^BN+N was extended by 50 days (Figure 5E), which demonstrated clear survival benefits over the other treatment groups. This survival advantage strongly correlated with the aforementioned tumor growth inhibition results. Together, these findings demonstrate that M@PLGA‐^10^BN‐mediated BNCT effectively inhibited tumor growth, thereby improving overall survival.

Based on the significant tumor‐suppressive effects observed, we evaluated post‐treatment tumor proliferation and apoptosis using histopathological analyses. A systematic assessment was performed using multiple methods including H&E staining, Ki‐67 immunohistochemistry, γ‐H2AX, and TUNEL staining. H&E staining revealed that the M@PLGA‐^10^BN+N treatment group exhibited a well‐defined boundary between the tumor tissue and normal brain tissue, with a significantly reduced tumor area compared to the other control groups (Figure 5G). High‐magnification observations demonstrated that the tumor cell nuclei in this group displayed typical apoptotic features, including blurred boundaries and fragmentation (Figure S11), whereas the other groups maintained intact cellular morphology and nuclear structure. This finding was consistent with the TUNEL staining results, which showed a significantly elevated rate of apoptosis in the M@PLGA‐^10^BN+N group (Figure S11). Immunofluorescence analysis indicated that the expression of the proliferation marker Ki‐67 was significantly reduced in the M@PLGA‐^10^BN+N group, whereas the fluorescence signal of the apoptosis‐related protein Caspase‐3 was the strongest. In contrast, the control groups exhibited high Ki‐67 and low Caspase‐3 expression patterns, suggesting sustained tumor cell proliferation with limited apoptosis (Figure 5H; Figure S12A). γ‐H2AX is recognized as a marker of DNA double‐strand breaks (DSBs) induced by radiotherapy. To confirm DSBs triggered by NPs‐mediated BNCT, we performed γ‐H2AX immunofluorescence analysis, which demonstrated that the γ‐H2AX signal intensity in the M@PLGA‐^10^BN+N group was significantly higher than in other groups (Figure 5I; Figure S12B). These results verify that M@PLGA‐^10^BN, when combined with neutron irradiation, induces DSBs in vivo.

Finally, we evaluated the biosafety and effects of BNCT with both types of NPs on normal tissues. Comprehensive assessments included biochemical analysis and H&E staining of heart, liver, spleen, lung, and kidney tissues. The levels of aspartate aminotransferase (AST), alanine aminotransferase (ALT), blood urea nitrogen (BUN), and creatinine (CRE) were within the normal ranges compared to those in the PBS groups (Figure S13A,B). H&E staining further confirmed the absence of obvious tissue lesions in any of the major organs (Figure S13C). To further objectively assess the immunogenicity of M@PLGA‐^10^BN, we measured the serum levels of IL‐6 and TNF‐α in mice at different time points post‐injection (Figure S14A). The experimental results demonstrated that, at various time points following nanoparticle injection, no significant elevation in IL‐6 or TNF‐α levels was observed in the M@PLGA‐^10^BN group compared with the control group, indicating that this biomimetic nanoparticle did not elicit a marked systemic inflammatory response (Figure S14B,C). Collectively, these findings demonstrate that both NPs exhibit excellent safety profiles without inducing long‐term toxic effects when BNCT is performed.

### 2.6 Nanocomposite‐Mediated BNCT Remodels the Tumor Immune Microenvironment In Vivo

**FIGURE 6:**
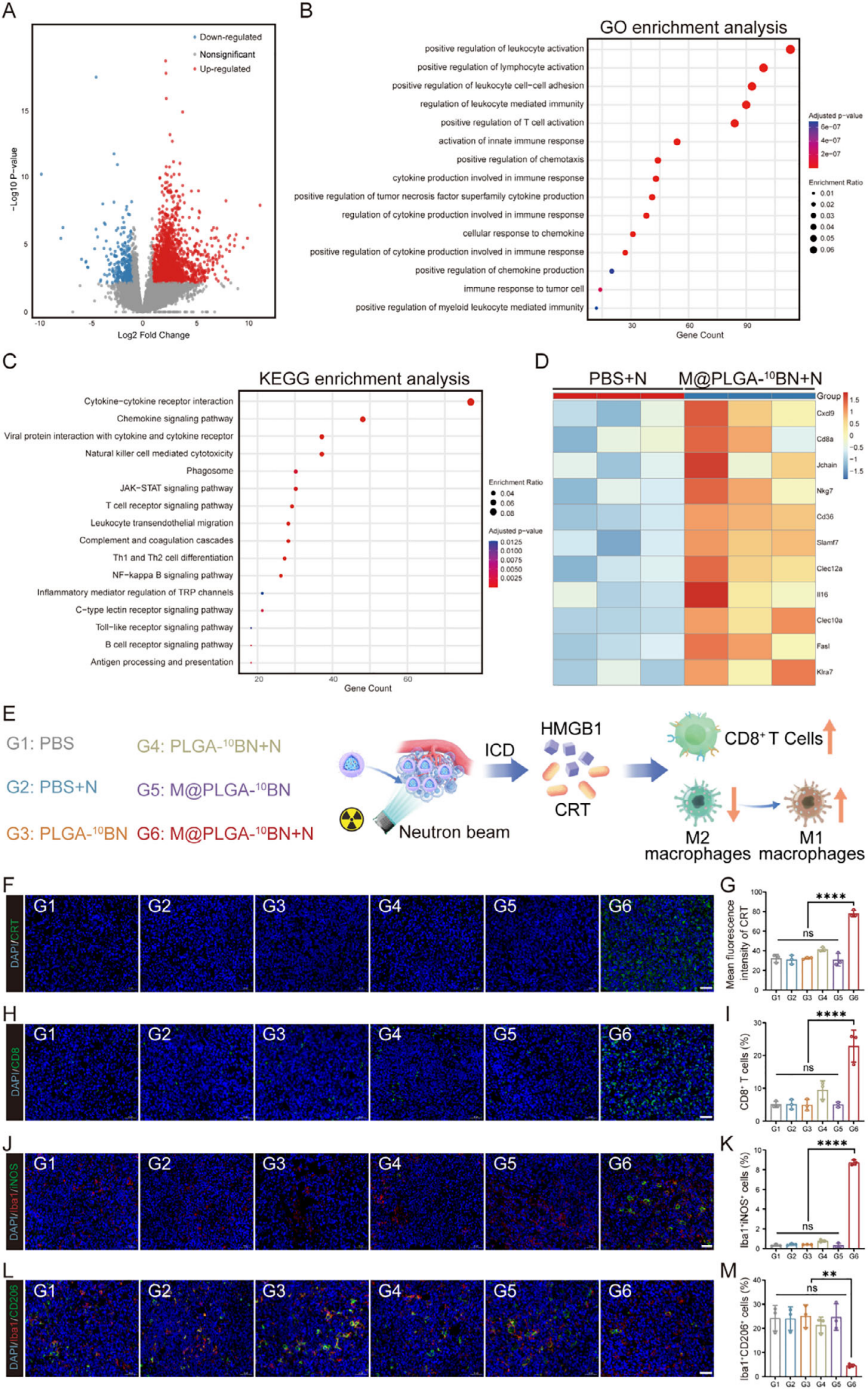
Nanocomposite‐mediated BNCT remodels the tumor immune microenvironment in vivo. (A) Differential gene‐distribution volcano plots between the PBS+N and M@PLGA‐^10^BN+N groups. (B) GO analysis of the PBS+N and M@PLGA‐^10^BN+N groups. (C) KEGG enrichment analysis in the PBS+N and M@PLGA‐^10^BN+N groups. (D) Heatmap of the differential expression genes related to immune response in the PBS+N and M@PLGA‐^10^BN+N groups. (E) Regulation of immune cells by M@PLGA‐^10^BN+N combined with neutron irradiation. (F and J) Representative immunofluorescence images of the expression of CRT in the glioma region after different treatments and the corresponding results of the semi‐quantitative analysis. Scale bar = 50 µm. (H and I) Representative immunofluorescence images of the degree of CD8^+^ T cell infiltration in tumor sections from different groups and the corresponding results of the semi‐quantitative analysis. Scale bar = 50 µm. (J and K) Representative immunofluorescence staining of iNOS (green) and Iba1 (red) in tumor sections from different groups and the corresponding results of the semi‐quantitative analysis. Scale bar = 50 µm. (L and M) Representative immunofluorescence staining of CD206 (green) and Iba1 (red) in tumor sections from different groups and the corresponding results of the semi‐quantitative analysis. Scale bar = 50 µm. Data presented are the mean ± SD. Statistical significance was calculated via one‐way ANOVA with Tukey's test: ns: non‐significant, ^*^ *p*<0.05, ^**^ *p*<0.01, ^***^ *p*<0.001, ^****^ *p*<0.0001.

Based on the in vitro results, it was demonstrated that M@PLGA‐^10^BN‐mediated BNCT directly triggered tumor cell apoptosis by inducing DNA damage while simultaneously promoting the release of tumor‐associated antigens. This suggests that the M@PLGA‐^10^BN‐mediated BNCT has the potential to remodel the immune microenvironment. To gain insight into the BNCT‐triggered changes in biological processes and immunological characteristics, we performed a transcriptomic analysis of tumor tissues from both the PBS+N and M@PLGA‐^10^BN+N groups. Transcriptomic analysis revealed 2,270 differentially expressed genes (DEGs) in the M@PLGA‐^10^BN+N group compared to the PBS+N group, among which 1,907 genes were significantly upregulated, and 363 genes were significantly downregulated (Figure 6A). Furthermore, KEGG and GO enrichment analyses demonstrated that the upregulated genes were primarily enriched in immune response‐related pathways, including innate immune activation, adaptive immune regulation, and inflammatory cytokine signaling (Figure 6B,C). Gene expression analysis was subsequently conducted for the two groups of samples. Notably, the expression of multiple immune‐related genes (Cxcl9, Cd8a, Cd36, J‐chain, and Nkg7) was significantly elevated in the M@PLGA‐^10^BN+N group (Figure 6D). These results indicated the enhancement of the antitumor immune response of M@PLGA‐^10^BN+ N‐mediated BNCT.

Building upon these transcriptomic insights, we further evaluated the dynamic changes in immune cells in the tumor microenvironment after treatment. Immunofluorescence results demonstrated that compared with other groups, the M@PLGA‐^10^BN+N treatment group exhibited significant upregulation of CRT expression in tumor tissues, along with prominent nucleocytoplasmic translocation of HMGB1 (Figure 6F,G; Figure S15). These changes confirm that BNCT effectively induces immunogenic cell death. Immune cell infiltration in post‐treatment tumor tissues was assessed to further analyze the remodeling of the tumor immune microenvironment. In the M@PLGA‐^10^BN+N group, the fluorescence intensity of CD8^+^ T cells significantly increased (Figure 6H,I), indicating that BNCT could enhance antitumor T cell immunity. Furthermore, fluorescence staining analysis was performed to determine the macrophage phenotype at the tumor site. The expression of inducible nitric oxide synthase (iNOS), a proinflammatory M1 macrophage marker, was significantly upregulated in the M@PLGA‐^10^BN+N group, whereas the expression of CD206, an anti‐inflammatory M2 macrophage marker, was markedly reduced (Figure 6J–M). In conclusion, our results demonstrated that M@PLGA‐^10^BN combined with BNCT not only induced immunogenic cell death but also significantly enhanced CD8^+^ T cell infiltration and promoted M1 macrophage polarization, and thereby synergistically improved the immunosuppressive microenvironment in glioma (Figure 6E).

### 2.7 Efficacy of Combined BNCT and Immune Checkpoint Blockade Evaluation In Vivo

**FIGURE 7:**
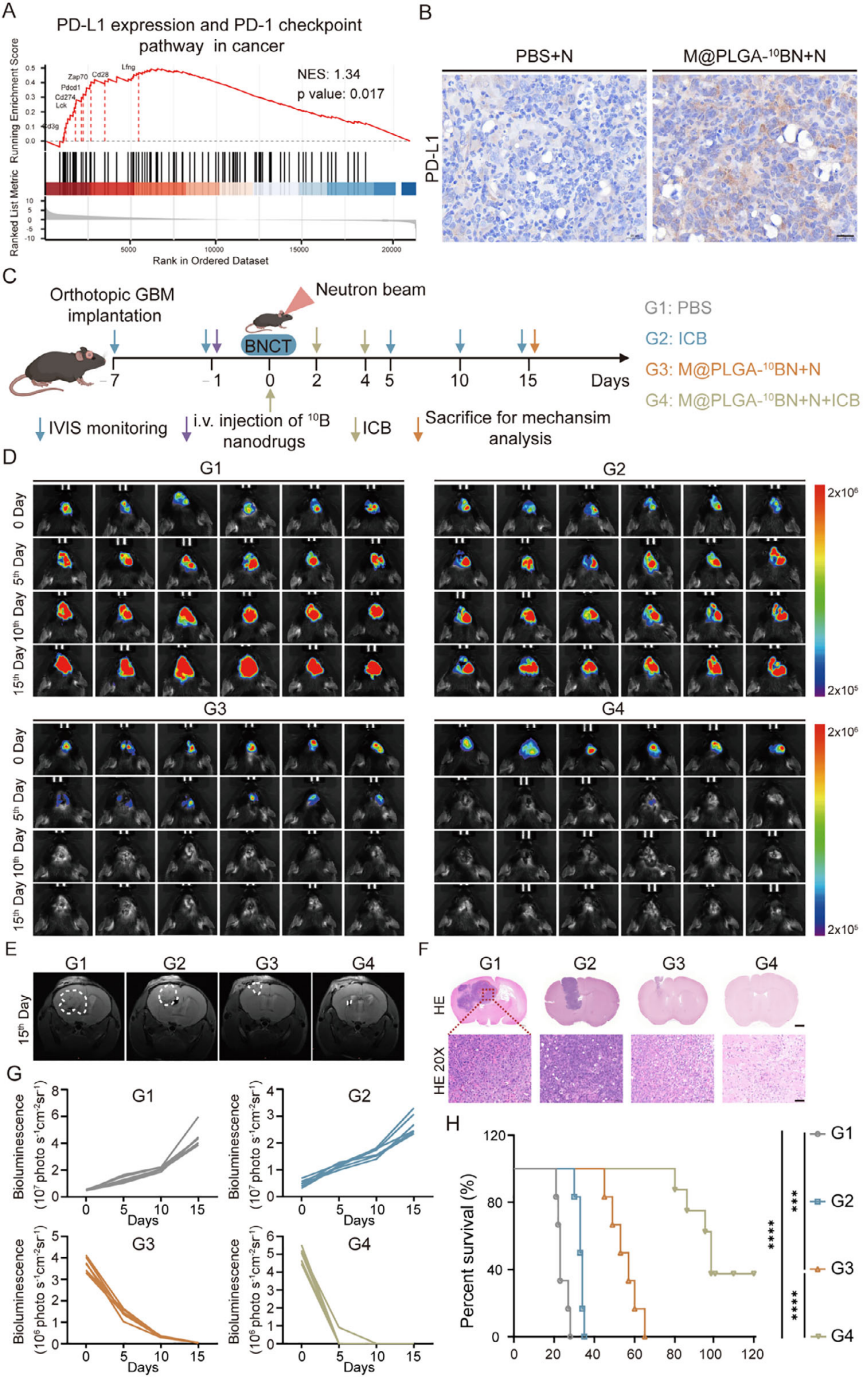
The efficacy of combined BNCT and immune checkpoint blockade (ICB) evaluation in vivo. (A) GSEA of PD‐L1 expression and PD‐1 checkpoint pathway for PBS+N against M@PLGA‐^10^BN+N (p = 0.017, n = 3). (B) Immunohistochemical analysis of PD‐L1 expression in tumor sections from PBS+N and M@PLGA‐^10^BN+N groups. Scale bar = 20 µm. (C) Schematic diagram of the therapeutic strategy. (D) Real‐time bioluminescent fluorescence images of mice bearing orthotopic GL261‐Luc glioma tumor at various timepoints after neutron irradiation in different groups (n = 6). (E) Representative MRI images on the 15th day after neutron irradiation in different groups. (F) H&E staining images in tumor tissues from GL261‐Luc glioma tumor‐bearing mice in different groups. Scale bar = 1000 µm, Scale bar = 50 µm. (G) Relative fluorescence intensity of each tumor‐bearing mice in each group (n = 6). (H) Kaplan−Meier survival analysis of GL261‐Luc glioma tumor‐bearing mice after neutron irradiation in different groups. Data presented are the mean ± SD. Statistical significance was calculated via one‐way ANOVA with Tukey's test: ns: non‐significant, ^*^ *p*<0.05, ^**^ *p*<0.01, ^***^ *p*<0.001, ^****^ *p*<0.0001.

The PD‐1/PD‐L1 signaling pathway—a critical adaptive immune evasion mechanism—negatively regulates the antitumor function of cytotoxic T cells via specific binding between PD‐L1 on tumor cell surfaces and PD‐1 receptors on activated T cells [27]. Gene enrichment analysis of the PD‐1/PD‐L1 checkpoint pathway at tumor sites revealed that this pathway was significantly upregulated following treatment with M@PLGA‐^10^BN combined with neutron irradiation, including the upregulation of the expression of genes related to this pathway (Lck, Cd3g, Lfng, and Zap70) (Figure 7A). Immunohistochemical analysis further confirmed the markedly elevated PD‐L1 protein expression levels in tumor tissues from the M@PLGA‐^10^BN+N group (Figure 7B). This finding was consistent with the Western blot results (Figure S16A,B). These findings suggest that this pathway may concurrently activate immunosuppressive feedback mechanisms, potentially compromising the tumor‐clearing capacity of cytotoxic T cells.

To systematically investigate the synergistic antitumor effects of M@PLGA‐^10^BN mediated BNCT combined with immune checkpoint blockade (ICB) therapy, we established an orthotopic glioma model using GL261‐Luc cells. As illustrated in Figure 7C, tumor‐bearing mice were randomly divided into four groups on day 7: PBS group, ICB group, M@PLGA‐^10^BN+N group (^10^B, 50 mg/kg body weight), and M@PLGA‐^10^BN+N+ICB group (^10^B, 50 mg/kg body weight). Neutron irradiation was performed 24 h after intravenous administration, with the combination therapy group receiving anti‐PD‐1 monoclonal antibody treatment (5 mg/kg body weight, every other day for three doses) starting on day 2 post‐irradiation. IVIS bioluminescence imaging demonstrated a time‐dependent decrease in the tumor signal intensity in both the M@PLGA‐^10^BN+N and combination therapy groups, with complete signal disappearance observed in the combination group by day 5, whereas the control group exhibited continuous signal enhancement (Figure 7D,G). MRI evaluation on day 15 further confirmed a significant reduction in the tumor volume in the M@PLGA‐^10^BN+N and combination therapy groups, with the latter demonstrating superior tumor suppression. In contrast, the ICB monotherapy and PBS groups exhibited marked tumor progression (Figure 7E). These findings demonstrated that ICB significantly enhanced the antitumor efficacy of M@PLGA‐^10^BN‐mediated BNCT. Survival analysis revealed prolonged survival in both the M@PLGA‐^10^BN+N and combination therapy groups, with surviving mice still observed in the combination group after 120 days (Figure 7H). H&E staining revealed well‐demarcated tumor‐normal brain tissue boundaries and significantly reduced tumor areas in the M@PLGA‐^10^BN+N and M@PLGA‐^10^BN+N+ICB groups (Figure 7F). High‐magnification microscopy revealed typical apoptotic features (nuclear fragmentation and indistinct borders) in the M@PLGA‐^10^BN+N group, whereas the combination group exhibited a near‐complete absence of intact tumor cell nuclei. In contrast, the control group maintained an intact cellular morphology and unbroken nuclei (Figure 7F). These results demonstrate that PD‐1 blockade significantly enhanced the therapeutic efficacy of M@PLGA‐^10^BN‐mediated BNCT.

### 2.8 Mechanism of Combination Therapy In Vivo

**FIGURE 8:**
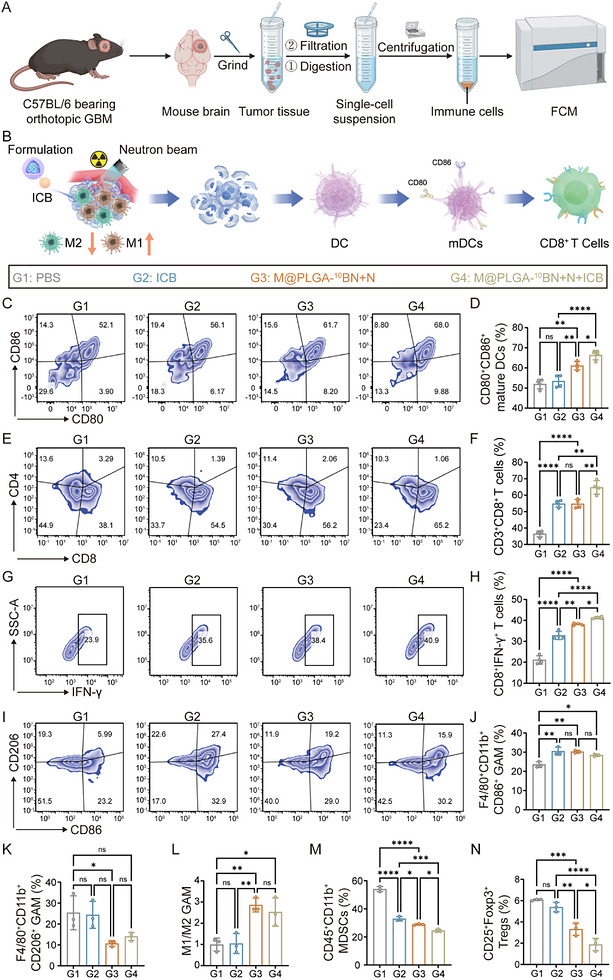
Mechanism of combination therapy in vivo. (A) Schematic illustration of brain tissue flow cytometry‐based analysis in C57BL/6 mice bearing orthotopic glioma. (B) Regulation of immune cells by BNCT plus ICB therapy. (C and D) Flow cytometry‐based analysis of intratumoral DC maturation and quantitative analysis of the proportion of intratumoral DC maturation (n = 4). (E and F) Flow cytometry‐based analysis of intratumoral CD8^+^ and CD4^+^ T cells and quantitative analysis of the proportion of intratumoral CD8^+^ T cells (n = 4). (G and H) Flow cytometry‐based analysis of intratumoral CD8+ IFN‐γ^+^ T cells and quantitative analysis of CD8^+^ IFN‐γ^+^ T cells (n = 4). (I–L) Flow cytometry‐based analysis of intratumoral M1‐type and M2‐type macrophages and quantitative analysis of the proportion of intratumoral M1‐type and M2‐type macrophages (n = 3). (M) Quantitative analysis of the proportion of intratumoral MDSCs in the brain of tumor‐bearing mice after different treatments (n = 3). (N) Quantitative analysis of the proportion of intratumoral Treg cells in the brain of tumor‐bearing mice after different treatments (n = 3). Data presented are the mean ± SD. Statistical significance was calculated via one‐way ANOVA with Tukey's test: ns: non‐significant, ^*^ *p*<0.05, ^**^ *p*<0.01, ^***^ *p*<0.001, ^****^ *p*<0.0001.

To explore the changes in the immune response caused by combination therapy. Tumor tissues were harvested from the mice on day 15 after treatment. Single‐cell suspensions were prepared by mechanical dissociation and enzymatic digestion, followed by immune cell enrichment via density gradient centrifugation. A comprehensive analysis of immune cell composition and activation status in the glioma microenvironment was performed using multicolor flow cytometry (Figure 8A). Flow cytometry‐based analysis revealed that both the M@PLGA‐^10^BN+N and M@PLGA‐^10^BN+N+ICB combination therapy groups exhibited significantly increased infiltration of mature dendritic cells (DCs, CD80^+^CD86^+^) in tumor tissues (61.1% and 66.3%, respectively) compared to the PBS control group (48.6%) and ICB monotherapy group (53.4%) (Figure 8C,D; Figure S17A). Notably, the M@PLGA‐^10^BN+N+ICB combination group demonstrated a significantly higher mature DC induction than the M@PLGA‐^10^BN+N monotherapy group (Figure 8C,D; Figure S17A), indicating that ICB further enhanced DC maturation and activation. Subsequent evaluation of CD3^+^CD8^+^ T cell proportions showed 54.77% in the ICB group and 54.75% in the M@PLGA‐^10^BN+N group, whereas the M@PLGA‐^10^BN+N+ICB combination group reached 64.72%, substantially exceeding the PBS control (36.47%) (Figure 8E,F; Figure S17B). In ICB therapy, increased CD8^+^IFN‐γ^+^ T cells are typically associated with improved therapeutic outcomes. Therefore, we further quantified CD8^+^IFN‐γ^+^ T cell populations within tumor tissues. Compared with the PBS group and ICB monotherapy group, both the M@PLGA‐^10^BN+N treatment group and M@PLGA‐^10^BN+N+ICB combination therapy group demonstrated significant increases in the infiltration of CD8^+^IFN‐γ^+^ T cells within tumor tissues (Figure 8G,H; Figure S17C). Notably, the M@PLGA‐^10^BN+N+ICB combination therapy induced a further statistically significant elevation in CD8^+^IFN^−^γ^+^ T cell populations compared to M@PLGA‐^10^BN+N monotherapy (Figure 8G,H; Figure S17C), suggesting that ICB administration provides additional enhancement of CD8^+^ T cell‐mediated anti‐tumor immune response. In addition, M@PLGA‐^10^BN+N treatment significantly promoted the transition from immunosuppressive M2‐type to antitumor M1‐type TAMs, increasing the M1 proportion from 23.53% to 30.23% while decreasing M2 proportion from 25.36% to 10.67%. Although similar M1/M2 ratio alterations were observed in the M@PLGA‐^10^BN+N+ICB combination group (28.43% vs. 14.03%), no significant difference was detected compared to the M@PLGA‐^10^BN+N group (Figure 8I–L; Figure S17D). These results indicate that M@PLGA‐^10^BN effectively promoted M1 macrophage polarization while suppressing the M2 phenotype, whereas the ICB combination primarily enhanced the T cell‐mediated immune response with a relatively limited regulatory role in macrophage polarization (Figure 8B). Finally, we analyzed the immunosuppressive cell populations in tumor tissues. Flow cytometry revealed that both M@PLGA‐^10^BN+N and M@PLGA‐^10^BN+N+ICB groups significantly reduced the infiltration of myeloid‐derived suppressor cells (MDSCs, CD45^−^CD11b^+^) compared to PBS and ICB groups, decreasing to 28.90% and 24.46%, respectively (Figure 8M; Figure S17E; Figure S18A). Similarly, regulatory T cell (Treg, CD25^+^Foxp3^+^) infiltration was markedly reduced in the treatment groups (Figure 8N; Figure S17F; Figure S18B). These data demonstrated that M@PLGA‐^10^BN+N therapy effectively suppressed the accumulation of immunosuppressive cells, including MDSCs and Tregs, in the tumor microenvironment, with potentially enhanced effects when combined with ICB treatment.

To investigate the effects of BNCT and combination therapy on peripheral immune organs, spleen tissues were collected from mice 15 days post‐treatment. Flow cytometry was used to analyze changes in various immune cell subsets within the spleen. The results demonstrated that both the M@PLGA‐^10^BN+N and M@PLGA‐^10^BN+N+ICB groups exhibited a significant increase in the number of mature dendritic cells (DCs, CD80^+^CD86^+^) compared with the PBS and ICB groups. However, no statistically significant difference was observed in mature DC counts between the combination and M@PLGA‐^10^BN+N groups (Figure S19A,B; Figure S20A). Notably, ICB failed to increase the number of mature DCs in the spleen, suggesting that the therapeutic effect of ICB may primarily involve modulation of the local TIM rather than exerting a regulatory influence on DC expansion in peripheral lymphoid organs (Figure S19A,B; Figure S20A). Flow cytometry‐based analysis of splenic CD3^+^CD8^+^ T cells revealed that ICB treatment significantly increased the population of this cell subset. Although an increasing trend in the number of CD3^+^CD8^+^ T cells was observed in the M@PLGA‐^10^BN+N treatment group, this change was not statistically significant. Notably, the M@PLGA‐^10^BN+N+ICB combination therapy group exhibited a significantly higher number of CD3^+^CD8^+^ T cells than the other treatment groups (Figure S19C,D; Figure S20B). Furthermore, the quantity of CD8^+^IFN‐γ^+^ T cells was markedly elevated in the M@PLGA‐^10^BN+N+ICB combination group relative to other groups (Figure S19E,F; Figure S20C). Our study revealed an intriguing phenomenon within the tumor microenvironment, neither in the M@PLGA‐^10^BN+N group nor in the combination therapy group, and the results demonstrated no significant alterations in CD4^+^ T cell infiltration. However, a distinct pattern of effects was observed in the peripheral immune organs. The proportion of CD4^+^ T cells increased significantly after M@PLGA‐^10^BN+N treatment, whereas the combination therapy failed to elicit a comparable effect (Figure S19C, M; Figure S20B). However, the mechanisms underlying this phenomenon require further investigation. Flow cytometric analysis revealed that M@PLGA‐^10^BN+N+ICB combination therapy exerted unique immunomodulatory effects. Compared with the control and other treatment groups, this combined regimen significantly reduced the proportion of immunosuppressive M2 macrophages while augmenting the population of immunostimulatory M1 macrophages. Notably, no significant changes in the M1/M2 macrophage ratio were observed in the spleen in the other groups (Figure S19G,H,N,O; Figure S20D). This finding demonstrates that only the combined application of BNCT and ICB can effectively remodel the polarization state of splenic macrophages, and that this systemic immunomodulatory effect may serve as one of the key mechanisms underlying its enhanced antitumor efficacy. Through a systematic analysis of splenic immunosuppressive cell populations, we observed the differential regulatory characteristics of MDSCs and Tregs following different treatments. Flow cytometry results revealed that, compared with the PBS group, both ICB and M@PLGA‐^10^BN+N treatments significantly reduced the proportion of splenic MDSCs, while the M@PLGA‐^10^BN+N+ICB combination therapy group exhibited more significant synergistic effects, with MDSC suppression markedly superior to the other groups (Figure S19I, J; Figure S20E). Regarding Treg cells, both the M@PLGA‐^10^BN+N treatment group and the M@PLGA‐^10^BN+N+ICB combination group showed significant inhibitory effects compared with the PBS and ICB groups, although no statistical difference was observed between these two groups (Figure S19K,L; Figure S20F). These results indicate that BNCT therapy effectively ameliorates systemic immunosuppression, while the addition of ICB selectively enhances MDSC suppression, but may have reached a plateau effect in Treg regulation, suggesting complementary roles of these two therapeutic approaches in modulating different immunosuppressive cell subsets. Collectively, these findings confirmed that combination therapy can reverse the immunosuppressive microenvironment and enhance the antitumor immune response.

## 3 Discussion

BNCT—a targeted alpha‐particle radiotherapy that exerts selective cytotoxic effects on tumor cells—was officially approved for clinical use in Japan in 2021. However, its clinical efficacy in glioma treatment remains not satisfactory [28]. The BBB restricts the delivery of existing boron‐containing compounds, limiting their ability to target tumors and penetrate deep into tumor tissues [29, 30]. Consequently, the development of novel boron‐based compounds and advanced delivery systems has emerged as a critical research focus. Although larger BN NPs can leverage the EPR effect for passive tumor targeting, their capacity to cross the BBB and penetrate deeply into tumor tissues remains constrained [9, 18]. Recent progress in nanotechnology has opened new avenues for efficient boron delivery, with studies demonstrating that boron‐containing nanoparticles exhibit promising tumor suppression in animal models [31, 32]. Among various nanocarriers, FDA‐approved PLGA has emerged as an ideal drug carrier because of its favorable safety profile and material properties. However, its clinical potential is limited by its rapid macrophage‐mediated clearance, which restricts its targeting efficiency in vivo [10, 33].

Cell membrane‐biomimetic drug‐delivery systems have been developed to address these challenges. These systems leverage the biocompatibility, immune evasion capability, and precise targeting properties of natural cell membranes to significantly enhance the therapeutic outcomes [15, 34]. In particular, macrophage membrane‐coated nanoparticles not only preserve the native protein and receptor structures but also improve tumor‐targeting efficiency while avoiding immune clearance [12]. Therefore, in this study, we designed a novel macrophage membrane‐wrapped polymeric nanoparticle system designated M@PLGA‐^10^BN. Compared with the conventional boron compounds boronophenylalanine (BPA) and sodium borocaptate (BSH), M@PLGA‐^10^BN demonstrated superior tumor accumulation and retention. ICP‐MS analysis revealed that the boron content of M@PLGA‐^10^BN in tumor tissue peaked at 53.15 µg/g 24 h post‐injection, with favorable tumor‐to‐normal tissue (T/N) and tumor‐to‐blood (T/B) ratios of 3.95 and 3.85, respectively (Figure 3G,H), which fully meets the therapeutic requirements for BNCT. Furthermore, this biomimetic nanoparticle exhibited significantly prolonged retention in tumor tissues, providing an extended irradiation time window for clinical applications.

Numerous studies have confirmed that BNCT induces tumor cell apoptosis through DNA damage [35, 36]. In mechanistic studies, our in vitro and in vivo experiments demonstrated that BNCT exerts cytotoxic effects on gliomas and suppresses tumor growth by inducing DNA damage and γ‐H2AX foci formation, thereby prolonging the survival of tumor‐bearing mice. However, whether BNCT can exert long‐term anti‐glioma effects through modulation of the immune microenvironment remains unclear. Therefore, this study investigated alterations in the glioma immune microenvironment following BNCT treatment. RNA sequencing and immunofluorescence analyses revealed that BNCT could reverse the immunosuppressive microenvironment, as evidenced by the increased infiltration of CD8^+^ T cells and M1 macrophages and the reduced population of immunosuppressive cells at the tumor sites (Figure 6H–M). Notably, our findings demonstrate that BNCT modulates immune cell populations within the splenic tissues. However, we also observed that BNCT concurrently activated the PD‐1/PD‐L1 pathway, potentially attenuating the antitumor function of T cells via immunosuppressive feedback mechanisms (Figure 7A, B). Currently, research on the synergistic effects of BNCT combined with ICB for glioma treatment remains limited. Consequently, we investigated a combination therapeutic strategy for BNCT using PD‐1 antibodies. The results demonstrated that the combined treatment not only exhibited superior efficacy compared to BNCT but also significantly improved the immune microenvironment, increased the population of immune cells, and reduced the proportion of immunosuppressive cells in the tumor and spleen (Figures 7, 8). These findings provide a critical rationale for the combined application of BNCT and other therapies and their subsequent clinical translation.

In recent years, hexagonal boron nitride (BN) nanoparticles have seen increasing applications in the biomedical field, and their biosafety has attracted widespread concern. Gianni Ciofani et al. evaluated the toxicity of hexagonal boron nitride nanotubes (BNNTs) on human endothelial cells and neuron‐like cells, demonstrating that BNNT concentrations up to 20 µg/mL exhibited good safety profiles for both cell types [37]. Kar et al. further confirmed through in vivo experiments that intravenous injection of h‐BN nanoparticles at doses ranging from 50 to 800 µg/kg in Wistar albino rats resulted in no significant changes in hematological and biochemical parameters compared with the control group, indicating no obvious toxicity within this dose range [38]. Notably, hexagonal boron nitride itself possesses excellent chemical inertness and thermal stability, and its toxicity is primarily dependent on factors such as nanoparticle morphology, size, surface modification, exposure dose, and duration [39]. Studies have shown that functional group modification can improve the chemical properties and hydrophilicity of BN nanoparticles, thereby enhancing their biocompatibility. Compared with unmodified BN nanoparticles, folic acid‐modified BN nanoparticles required higher concentrations to exert cytotoxicity against human dermal fibroblasts (HDFn) [40]. Furthermore, erythrocyte membrane‐coated BN exhibited no obvious toxicity in vitro against human embryonic kidney 293 (HEK293) cells or cervical cancer cells [41]. Further studies evaluating the acute and subacute toxicity of erythrocyte membrane‐coated BN nanosheets via intravenous injection revealed that significant acute toxicity was observed only at injection doses exceeding 168.07 mg/kg, whereas no obvious toxic effects were noted in subacute toxicity tests [42]. In our experiments, the injection dose was far below this value. Recent studies have shown that coating BN nanosheets with tumor cell membranes for boron neutron capture therapy exhibits favorable efficacy and safety [43]. Collectively, these studies suggest that biomimetic membrane‐coated BN nanomaterials (e.g., erythrocyte membrane or cancer cell membrane coating) can mimic natural cell membrane properties, thereby reducing immune recognition and clearance, enhancing cellular affinity, and improving biocompatibility. The overall toxicity of the M@PLGA‐^10^BN biomimetic nanoparticles constructed in this study, which incorporate PLGA polymers and macrophage membrane protein components, requires comprehensive evaluation. In our study, in vitro CCK‐8 assays showed that co‐incubation with GL261 cells at various concentration gradients for different time did not result in any significant decrease in cell viability (Figure S7A–D). In vivo experimental results demonstrated that H&E staining of major mouse organs revealed no obvious inflammatory infiltration or tissue damage during the treatment period. Serum biochemical parameters (ALT, AST, BUN, CRE) showed no significant differences compared with the control group (Figure S13A–C). In vivo immunogenicity evaluation further confirmed that the biomimetic nanoparticles did not induce a marked elevation in the levels of the pro‐inflammatory cytokines IL‐6 and TNF‐α (Figure S14A–C). Therefore, the experimental data from this study support the favorable biosafety profile of M@PLGA‐^10^BN under a single‐dose administration regimen. However, current toxicity studies on boron‐based nanomaterials still have certain limitations, such as insufficient human exposure data and unclear chronic in vivo toxicity, which require further elucidation.

Moreover, M@PLGA‐^10^BN demonstrated superior manufacturability compared with many boron compounds. Modular self‐assembly can be achieved through simple ultrasonication, offering a straightforward process with controllable costs and potential for large‐scale production. Specifically, this method features simple operation, low equipment requirements, and readily available PLGA [11, 33, 44, 45]. Furthermore, this nanocomposite exhibited high modifiability. This allows for the substitution of diverse boron compounds and the preparation of “customized” delivery via macrophage membrane modification to meet various clinical needs [46]. Nevertheless, limitations remain, including the high cost and time‐consuming nature of cell membrane scaling, residual organic solvents requiring additional purification, and broad nanoparticle size distribution that affects batch‐to‐batch reproducibility [44, 47]. Addressing these challenges will be critical for advancing the clinical translation of this biomimetic nanoplatform.

## 4 Conclusion

In this study, we successfully prepared macrophage membrane‐coated PLGA‐^10^BN nanoparticles, which demonstrated excellent biocompatibility and stability. The biomimetic nanoparticles effectively crossed the blood‐brain barrier and specifically accumulated in glioma tissue. At 24 h post‐intravenous injection, ICP‐MS analysis revealed a ^10^B concentration of 53.15 µg/g in tumor tissue, with both T/N and T/B ratios exceeding 3.5, meeting the requirements for BNCT. Both in vitro and in vivo experiments confirmed that this approach effectively induced DNA damage in tumor cells and significantly suppressed glioma growth. Further investigation revealed that, beyond direct tumor cell killing, the therapy could reverse the immunosuppressive tumor microenvironment. When combined with a PD‐1 blockade, enhanced antitumor efficacy and synergistic immunomodulatory effects were observed, particularly demonstrating potential in activating systemic antitumor immunity. Overall, M@PLGA‐^10^BN is expected to be used as an ideal boron delivery agent for BNCT in gliomas owing to its exceptional targeting capability; Moreover, it may also represent a versatile nanocomposite for the delivery of other boron compounds. In addition, BNCT combined with ICB offers a promising clinical approach for improving treatment efficacy in patients with glioma.

## 5 Experimental Section

### 5.1 Materials

The boron‐10 enriched boric acid (abundance of 99.43%) was purchased from Liaoning Honghao Chemical Industry Co., Ltd., and melamine (catalog number: M108433) was supplied by Shanghai Aladdin Biochemical Technology Co., Ltd. (Shanghai, China). Brefeldin (HY‐16592), chloroquine (MCE, HY‐17589A), chlorpromazine (MCE, HY‐12708), and colchicine (MCE, HY‐16569) were obtained from MedChemExpress (Shanghai, China). Filipin (MellonBio, MB1848) was obtained from Meilun Bio (Dalian, China). Poly(lactic‐co‐glycolic acid) (PLGA, 50:50‐COOH, 15 kDa) was obtained from Jinan Daigang Biotechnology Co., Ltd. Beyotime Biotechnology provided DiD lipophilic tracer (catalog number: C1039), CCK‐8 cell proliferation kit (catalog number: C0037), apoptosis detection kit (catalog number: C1062S), cell cycle analysis kit (catalog number: C1052), DAPI staining solution (Catalog number: C1006), crystal violet staining solution (catalog number: C0121), Hoechst 33258 staining solution (catalog number: C1017), Lyso‐Tracker Green (catalog number: C1047S) and phosphate‐buffered saline (PBS, catalog number: C0221B). Electron microscope fixative (catalog number: BL911A) was obtained from Biosharp Life Sciences. Cell culture reagents, including Dulbecco's Modified Eagle Medium (DMEM, catalog number: A5669701), fetal bovine serum (FBS, catalog number: A5669701), penicillin–streptomycin solution (catalog number: 15140122), and 0.25% trypsin‐EDTA (catalog number: 25200072), were purchased from Gibco.

### 5.2 Antibodies

The antibodies used in this study are summarized as follows: γ‐H2AX (catalog number: GB111841), integrin α4 (catalog number: 87464‐1‐RR), integrin β1 (catalog number: GB115173), CD47 (catalog number: A1838), CRT (catalog number: GB112134), CRT‐Alexa Fluor 488 (catalog number: 62304S), PD‐L1 (catalog number: GB155704), GAPDH (catalog number: GB15004), β‐tubulin (catalog number: GB1101), HMGB1 (catalog number: GB11103), cleaved caspase‐3 (catalog number: GB115733), Ki‐67 (catalog number: GB111141) from Wuhan Servicebio Technology Co., Ltd. CD4 (Abcam, catalog number: ab183685); CD8(Abcam, catalog number: ab217344); iba1 (Abcam, Catalog number: ab178846); iNOS (Abcam, catalog number: ab178945), CD86 (Abcam, catalog number: ab119857); C206 (Abcam, catalog number: ab64693); CD80‐PE‐Cyanine5 (eBioscience, catalog number: 15‐0801‐82); CD86‐PE (eBioscience, catalog number: 12‐0862‐82); F4/80‐eFluor 450 (eBioscience, catalog number: 48‐4801‐82); CD206‐Alexa Fluor 700 (eBioscience, catalog number: 56‐2061‐82); CD45‐APC (eBioscience, catalog number: 17‐0451‐82); CD3‐FITC (eBioscience, catalog number: 11‐0032‐82); CD8a‐PE(eBioscience, catalog number: 12‐0081‐82); CD4‐PerCP‐Cyanine5.5 (eBioscience, catalog number: 45‐0042‐82); CD11c‐FITC (eBioscience, catalog number: 11‐0114‐82); CD80‐Super Bright 436 (eBioscience, catalog number: 62‐0801‐82); CD11b‐Alexa Fluor 488 (eBioscience, catalog number: 53‐0112‐82); CD25‐Alexa Fluor 488 (eBioscience, catalog number: 53‐0251‐82); and Foxp3‐PE (eBioscience, catalog number: 12‐4774‐41).

### 5.3 Cell Culture and Animals

The cell lines used in the experiment and their culture conditions were as follows: The RAW264.7 macrophage cell line was purchased from Shanghai Zhongqiao Xinzhuo Biotechnology Co., Ltd. (catalog number: ZQ0098) and cultured in high‐glucose DMEM medium supplemented with 10% fetal bovine serum (FBS) and 1% penicillin–streptomycin at 37°C in a 5% CO_2_ incubator. The GL261 glioma cell line and its luciferase‐labeled strain GL261‐Luc were both obtained from Shanghai Zhongqiao Xinzhuo Biotechnology Co., Ltd. (catalog numbers: ZQ0932 and LZQ0065, respectively) and routinely cultured in high‐glucose DMEM medium containing 10% FBS under the same conditions (37°C, 5% CO_2_). All the cell culture procedures were performed under aseptic conditions. Six‐week‐old male C57BL/6 mice that were used in the study were provided by GemPharmatech Co. Ltd. (Jiangsu, China). All animal experiments were conducted in strict accordance with the ethical guidelines for laboratory animal use and approved by the Animal Ethics Committee of Fudan University (approval number: 2023‐HSYY‐480JZS). All mice were housed in specific pathogen‐free (SPF) environments with 12‐h light/dark cycles and were provided adequate food and water.

### 5.4 Cell Membrane Derivation

Macrophage membranes were extracted using an optimized protocol based on previously described methods as follows [48]: First, cells reaching 90% confluence were collected and washed with ice‐cold PBS three times, followed by centrifugation at 800 rpm for 5 min to remove supernatant. The cell pellet was resuspended in a membrane protein extraction buffer containing PMSF and incubated on ice for 15 min. Complete cell lysis was achieved through three freeze–thaw cycles alternating between −80°C and room temperature. The membrane fraction was subsequently isolated by centrifugation at 14 000 g for 30 min at 4°C. To obtain homogeneous membrane components, the crude membrane extract was passed through a 400 nm polycarbonate membrane in a mini‐extruder for a minimum of 21 passes to eliminate larger membrane fragments. The final membrane pellet was collected as pale white pellets and stored at −80°C. Before use, the membranes were resuspended in deionized water with mild sonication, and the membrane proteins were quantified using a standard BCA assay.

### 5.5 Synthesis of h‐^10^BN Nanoparticles

The h‐^10^BN was synthesized according to a previously reported method. The detailed experimental procedure is as follows: enriched ^10^B‐boric acid (0.366 g, 6 mmol) and melamine (0.126 g, 1 mmol) were precisely weighed and thoroughly ground in an agate mortar. The homogeneous mixture was then transferred to a corundum ark (60 mm × 30 mm × 15 mm). The corundum ark was placed in a horizontal tube furnace, where the nitridation process was carried out under continuous N_2_ flow (100 mL/min) with programmed heating at a rate of 10°C/min to 900°C, and then maintained at this temperature for 3 h. After cooling, an ivory‐white bulk product was obtained, which was ground into crude h‐^10^BN powder. The purification process consisted of the following steps. First, the nanoparticles were fully dispersed via water bath sonication for 4 h. Small‐molecule byproducts were then removed by aqueous dialysis for 48 h using a dialysis membrane with a molecular weight cutoff of 8–14 kDa. Finally, the suspension was centrifuged at 1811 g for 5 min to collect the milky supernatant containing h‐^10^BN nanoparticles. The purified product was lyophilized for 24 h and stored in a desiccator until subsequent use.

### 5.6 Preparation of PLGA Cores and M@PLGA‐^10^BN

The ^10^BN‐loaded PLGA NPs were prepared using a modified traditional oil‐in‐water (O/W) single‐emulsion solvent evaporation method [49]. Specifically, 50 mg PLGA was dissolved in 2 mL dichloromethane (DCM), and dissolution was accelerated by ultrasonication. After the complete dissolution of PLGA in DCM, 5 mg ^10^BN was added, followed by further ultrasonication to ensure a homogeneous dispersion of ^10^BN. The organic phase was emulsified using probe sonication at 63 W for 2 min (5 s on/5 s off) in an ice bath. Subsequently, 8 mL of a 4% polyvinyl alcohol (PVA) aqueous phase was added to the organic phase, and emulsification was continued at 52 W for 2 min (5 s on/5 s off) under ice‐cooling to form a uniform emulsion.

To solidify the NPs and evaporate residual DCM, 10 mL of a 2% isopropanol solution was added to the emulsion, followed by magnetic stirring at room temperature for 12 h. The nanoparticles were collected by centrifugation. This was followed by centrifugation at 2 500 rpm for 5 min, to remove excess PVA, and subsequently, at 11 000 rpm for 30 min to isolate the NPs. The harvested nanoparticles were thoroughly washed three times with deionized water and resuspended in deionized water. Furthermore, the DiD‐labeled PLGA NPs were prepared using a similar procedure, except that DiD was substituted for ^10^BN NPs.

For the preparation of M@PLGA‐^10^BN NPs, a previously reported sonication technique was employed. Briefly, collected macrophage membranes were suspended in deionized water and sonicated at 200 W for 3 min (5 s on/5 s off) in an ice bath to obtain macrophage membrane vesicles. Subsequently, the PLGA‐^10^BN NPs were mixed with the macrophage membrane vesicles at a weight ratio of 0.5:1 (membrane protein to PLGA). The mixture was then sonicated using the bath sonicator for 5 min, and ultimately resulted in the preparation of M@PLGA‐^10^BN. M@PLGA‐DiDs were prepared by using the same method.

### 5.7 Physicochemical Characterization of h‐^10^BN and M@PLGA‐^10^BN

The particle sizes and zeta potentials of h‐^10^BN, PLGA‐^10^BN, and M@PLGA‐^10^BN were precisely determined by dynamic light scattering (DLS) using a NanoZS instrument (Malvern Instruments, Malvern, UK). Powder X‐ray diffraction (XRD) patterns of h‐^10^BN were recorded on a Bruker D8 Advance diffractometer with Cu Kα radiation (λ = 1.5406 Å) operated at 40 kV and 40 mA. The Fourier‐transform infrared (FTIR) spectra of the h‐^10^BN powder were acquired in attenuated total reflectance (ATR) mode using a Nicolet iS50 spectrometer (Thermo Fisher Scientific, USA). Transmission electron microscopy (TEM) images of h‐^10^BN, PLGA‐^10^BN, and M@PLGA‐^10^BN were obtained using a Tecnai G2 Spirit BioTwin (JEM‐200CX, JEOL, Tokyo, Japan) field‐emission scanning electron microscope at an accelerating voltage of 100 kV. High‐resolution transmission electron microscopy (HRTEM) images were obtained using a JEOL JEM‐2100F field‐emission transmission instrument. All samples for electron microscopy observation were prepared by evaporating approximately 5 µL of the sample solution onto ultrathin carbon‐coated copper grids, followed by staining with 1% uranyl acetate for 30 s prior to imaging.

### 5.8 Identification of Membrane Associated Protein

To identify membrane‐associated proteins, macrophage lysates, purified macrophage membranes, M@PLGA‐^10^BN, and PLGA‐^10^BN were lysed in RIPA buffer (supplemented with 1% protease inhibitor and 1% phosphatase inhibitor) on ice for 15 min. The lysates were then centrifuged at 14 000 g for 10 min at 4°C. The resulting supernatant proteins were mixed with SDS loading buffer, heated at 100°C for 5 min, and loaded onto SDS‐polyacrylamide gel for electrophoresis. For total protein imaging, the gel was stained with Coomassie Blue fast staining solution and washed overnight with pure water before imaging using a ChemiDoc XRS^+^ system (Bio‐Rad). For Western blot analysis, proteins were transferred onto PVDF membranes, a protein‐free rapid blocking solution for 10 min at room temperature and incubated with primary antibodies against integrin α4 (1:1000), integrin β1 (1:10000), and CD47 (1:500). After three washes with TBST, the membranes were incubated with HRP‐conjugated goat anti‐rabbit secondary antibodies for 30 min at room temperature. Following three additional TBST washes, protein bands were visualized using ECL reagent and captured with the ChemiDoc XRS^+^ system.

### 5.9 Release Study of PLGA‐^10^BN and M@PLGA‐^10^BN

To evaluate the drug‐release profile of ^10^BN from the PLGA‐^10^BN and M@PLGA‐^10^BN NPs, the release rate of ^10^BN was investigated using the dialysis membrane method. The PLGA‐^10^BN and M@PLGA‐^10^BN NPs were redispersed in 5.0 mL of pH 7.4 PBS or pH 6.5 phosphate buffer. The suspension was placed in a dialysis tubing cellulose membrane (68035, molecular weight cutoff: 10,000 Da, Thermo Fisher Scientific) and added to 95 mL of pH 7.4 PBS or pH 6.5 phosphate buffer. The sample suspensions were shaken at 50 rpm at 37°C. After 0, 0.5, 1.0, 2.0, 4.0, 8.0, 12.0, 24, 48, and 72 h, the samples were extracted, and the amount of released ^10^BN content was quantitatively determined by inductively coupled plasma mass spectrometry (ICP‐MS).

### 5.10 Cellular Uptake Pathway Analysis

GL261 cells were seeded in a 12‐well plate (1 × 10^5^ cells/well) and allowed to adhere for 24 h. Subsequently, the cells were pre‐treated with various endocytosis inhibitors diluted in DMSO at 37°C for 60 min. The final concentrations of the inhibitors were as follows: 10 µM brefeldin A (MCE, HY‐16592), 10 µM chloroquine (MCE, HY‐17589A), 10 µg/mL chlorpromazine (MCE, HY‐12708), 10 µM colchicine (MCE, HY‐16569) or 10 µg/mL filipin (Mellonbio, MB1848). Following inhibitor treatment, all groups were washed once with PBS and incubated with M@PLGA‐DiD. After incubation, the culture medium was removed, and cellular uptake was quantified using flow cytometry.

### 5.11 Intracellular Fate of M@PLGA‐DiD

Briefly, GL261 cells were seeded in confocal dishes and cultured for 24 h to reach approximately 80% confluence. After incubation with the M@PLGA‐DiD for indicated time periods, cells were washed with PBS, followed by staining with Hoechst (5 µg/mL) and LysoTracker (50 nM) for 15 min at room temperature. The solution was then removed, and the cells were washed with PBS. Images were captured under a 60× oil immersion lens using confocal microscopy.

### 5.12 Cellular Uptake

Cellular uptake was evaluated by confocal laser scanning microscopy (CLSM) and flow cytometry. GL261 cells were seeded at an appropriate density into confocal‐specific dishes and 6‐well plates and cultured for 24 h to allow cell adhesion. Subsequently, PBS, PLGA‐DiD, and M@PLGA‐DiD were added at equal concentrations and incubated for 2, 4, 8, and 12 h. After incubation, the medium was removed, and the cells were washed three times with PBS to eliminate non‐internalized nanoparticles. For confocal microscopy observations, the cells were fixed with 4% paraformaldehyde at room temperature for 20 min, washed three times with PBS, stained with DAPI for 5 min to label nuclei, and then washed again with PBS before imaging under a confocal microscope to assess the intracellular distribution of NPs. For quantitative analysis by flow cytometry, the cells were trypsinized, collected by centrifugation at 800 rpm for 5 min, washed three times with PBS, and resuspended in an appropriate volume of PBS. The fluorescence intensity of cells was measured immediately using a flow cytometer.

For the CCK‐8 cytotoxicity assay, GL261 cells were seeded into 96‐well plates at a density of 5×10^3^ cells/well and cultured for 24 h. Subsequently, culture media containing PLGA‐^10^BN or M@PLGA‐^10^BN nanoparticles with varying ^10^B concentrations (0, 10, 50, 100, and 200 µg/mL) were added to the respective wells, followed by further incubation for 12, 24, 48, and 72 h. After removing the culture medium, the cells were washed three times with PBS. Then, 100 µL of fresh medium containing 10% CCK‐8 reagent was added to each well, and the plates were incubated at 37°C for 2 h. The absorbance was measured at 450 nm using a microplate reader, and the relative cell viability was calculated.

For quantitative analysis of boron content in vitro, GL261 cells were seeded in six‐well plates (1×10^5^ cells/well) and cultured for 24 h. Subsequently, PLGA‐^10^BN and M@PLGA‐^10^BN NPs with a ^10^B concentration of 100 µg/mL were added and incubated for an additional 24 h. The culture medium was discarded, and the cells were washed three times with PBS to completely remove residual NPs. Following this, the cells were digested with 0.25% trypsin, centrifuged at 800 rpm for 5 min to collect the cell pellet, washed twice with PBS, and resuspended in an appropriate volume of PBS. Cells were counted using an automated cell counter. For sample preparation, a mixed‐acid digestion method was employed: 4 mL concentrated nitric acid and 4 mL hydrofluoric acid were added, followed by sealed digestion at 230°C for 30 min. After cooling, the samples were transferred to a polytetrafluoroethylene (PTFE) beaker and heated at 200°C for 6 h to remove residual hydrofluoric acid. The ^10^B content of the solution was quantified by ICP‐MS.

### 5.13 Construction of In Vitro BBB Model

Transwell inserts with 3 µm pores (growth area 0.33 cm^2^) were pre‐coated with 2% gelatin. bEnd.3 cells were seeded at 1×10^4^ cells per insert into the upper chamber in 200 µL medium, while 800 µL medium was added to the lower chamber. The culture media in both compartments were refreshed every 48 h for 14 days.

### 5.14 In Vitro BBB Permeability Evaluation

GL261 cells were seeded in the lower chamber of a transwell system at a density of 1×10^5^ cells/well. After 24‐h incubation, equal concentrations of PLGA‐DiD and M@PLGA‐DiD suspensions were added to the upper chamber. The experiment was terminated after incubation at 37°C under 5% CO_2_ for 8, 12, and 24 h. The medium in the lower chamber was removed, followed by three washes with ice‐cold PBS. Subsequently, the cells were detached using 0.25% trypsin (containing 0.02% EDTA) at room temperature for 1 min. The cell suspension was centrifuged at 800 rpm for 5 min to obtain a cell pellet, which was then washed twice with PBS to remove unbound nanoparticles. Finally, the cells were resuspended in PBS, and the intracellular DiD fluorescence intensity was measured using flow cytometry.

To evaluate the ex vivo BBB penetration capacity of the PLGA‐^10^BN and M@PLGA‐^10^BN NPs, TEM was used to examine the intracellular distribution of both NPs within tumor cells. GL261 cells were seeded in the lower chamber of the transwell inserts at a density of 1×10^5^ cells/well. After 24‐h incubation to ensure complete cell adhesion, PLGA‐^10^BN and M@PLGA‐^10^BN NPs (each containing 100 µg/mL ^10^B) were added, followed by an additional 24‐h incubation. After treatment, the culture medium was discarded and the cells were gently washed three times with PBS (pH 7.4) to remove non‐internalized NPs. Subsequently, the cells were trypsinized using 0.25% trypsin, centrifuged at 800 rpm for 5 min to collect the cell pellets, and washed twice with PBS.

For TEM processing, cell pellets were resuspended in 1 mL freshly prepared 2.5% glutaraldehyde (in 0.1 M phosphate buffer, pH 7.4), fixed for 30 min at room temperature in the dark, and then fixed overnight at 4°C. Standard TEM sample preparation procedures were then performed, including post‐fixation with 1% osmium tetroxide, dehydration through an ethanol series, and embedding in epoxy resin. Ultrathin sections (70–90 nm) were prepared, double‐stained with uranyl acetate and lead citrate, and observed by TEM at an accelerating voltage of 120 kV.

### 5.15 Tumor Targeting Capability and Biodistribution of M@PLGA‐^10^BN In Vivo

The orthotopic glioma transplantation model was established by intracranial injection of 1×10^5^ GL261‐Luc cells (total volume: 5 µL) into the right striatum of mice (2 mm lateral to the midline, 2.5 mm depth). Seven days after the injection, robust bioluminescent signals from tumor cells were observed using the in vivo imaging system (IVIS), confirming the successful establishment of the model. Subsequently, free DiD, PLGA‐DiD, and M@PLGA‐DiD were administered via tail vein injection. To evaluate nanoparticle accumulation in the brain, dynamic monitoring of intracranial fluorescence signals was performed at 4, 8, 12, 24, and 48 post‐injection using the IVIS. At 24 h post administration, the mice were subjected to terminal anesthesia and perfusion, followed by the collection of major organ samples for ex vivo fluorescence quantification using the IVIS. Moreover, brain tissues were fixed in 4% paraformaldehyde (PFA) solution for 24 h for subsequent processing. Whole‐tissue section scanning was used to examine the distribution patterns of free DiD, PLGA‐DiD, and M@PLGA‐DiD within the brain parenchyma.

In the pharmacokinetic study, tumor‐bearing mice were intravenously injected via the tail vein with PLGA‐^10^BN and M@PLGA‐^10^BN (^10^B, 50 mg/kg body weight, n = 3 per group). At 12, 24, and 48 h post‐administration, the mice were euthanized, and major organs (heart, liver, spleen, lung, kidney, normal brain tissue, and tumor) and blood samples were rapidly collected for wet‐weight determination. The ^10^B content of these tissues and blood samples was quantitatively analyzed using ICP‐MS. The detailed operational procedure was as follows: The harvested organ tissues and blood samples were separately digested with 4 mL nitric acid and 4 mL hydrofluoric acid in a reaction vessel at 230°C for 30 min. After cooling, the digested solution was transferred to a polytetrafluoroethylene (PTFE) beaker and heated at 200°C for 6 h to remove residual hydrofluoric acid. Finally, the solution was transferred to a 100 mL volumetric flask, diluted, and thoroughly mixed before ^10^B content analysis.

To further investigate the intratumoral distribution of nanoparticles, tumor‐bearing mice were intravenously administered PLGA‐^10^BN and M@PLGA‐^10^BN (^10^B, 50 mg/kg body weight) via the tail vein. At 24 h post‐injection, euthanasia was performed, and the tumor tissues were promptly harvested. The residual blood on the tissue surface was rinsed off, and the samples were sectioned into rice grain‐sized pieces using a sharp blade. Subsequently, the specimens were fixed in 2.5% glutaraldehyde at room temperature under light‐protected conditions for 30 min, followed by additional fixation at 4°C for 24 h in the dark. TEM was used to analyze the intratumoral distribution of the NPs.

### 5.16 Efficacy Assessments of BNCT on GL261 Cells

2×10^6^ GL261 cells were seeded in 10 cm dishes and allowed to adhere for 24 h. The cells were then co‐incubated with different formulations (^10^B concentration: 100 µg/mL) at 37°C for 24 h. Following incubation, the cells were washed with PBS, detached by trypsinization, washed three times with PBS, and pelleted by centrifugation at 800 rpm for 5 min. Before neutron irradiation, cells were resuspended in 200 µL complete medium and transferred to centrifuge tubes. Some tubes were exposed to a neutron beam for 20 min (neutron flux: 6.96×10^8^ n cm^−2^ s^−1^) using the AB‐BNCT medical demonstration facility installed at Mazu Campus, Fujian Medical University Union Hospital, which was developed by Lanzhou University and possesses independent intellectual property rights. After irradiation, the cells were reseeded for subsequent in vitro efficacy assays.

### 5.17 Cell Viability Assay

Treated cells were centrifuged at 800 rpm for 5 min and washed three times with PBS. Subsequently, 4×10^3^ GL261 cells were seeded in a 96‐well plate and cultured for different time. After incubation, the culture medium was removed, and the cells were gently washed three times with PBS. Next, 100 µL of medium containing 10% CCK‐8 reagent was added to each well, followed by incubation at 37°C for 2 h. Finally, the absorbance of each well was measured at 450 nm using a microplate reader, and the relative cell viability of each experimental group was calculated.

### 5.18 Apoptosis Analysis

Post treatment, the cells were pelleted by centrifugation at 800 rpm for 5 min and washed three times with PBS. Subsequently, 2×10^5^ cells per well were seeded in 6‐well plates and cultured for an additional 48 h. After incubation, the medium was removed, and the cells were trypsinized, collected by centrifugation at 800 rpm for 5 min, and thoroughly washed three times with PBS. Finally, the cells were stained according to the manufacturer's protocol using an Annexin V‐FITC apoptosis detection kit, and apoptosis was analyzed by flow cytometry.

### 5.19 Cell‐Cycle Analysis

Post‐treatment, the cells were pelleted by centrifugation at 800 rpm for 5 min and washed three times with PBS. Subsequently, 2×10^5^ cells per well were seeded in 6‐well plates and cultured for an additional 48 h. After incubation, the medium was removed, and the cells were trypsinized, collected by centrifugation at 800 rpm for 5 min, and thoroughly washed three times with PBS. Finally, the cells were strictly stained according to the manufacturer's protocol for the cell‐cycle detection kit, and the cell cycle was measured by flow cytometry.

### 5.20 Clonogenic Assay

For this assay, 2×10^3^ treated GL261 cells were seeded into 6‐well plates and cultured for 10 days (37°C, 5% CO_2_). The medium was carefully aspirated, and the cells were washed three times with ice‐cold PBS. The cells were then fixed with 4% PFA solution at room temperature for 20 min. After fixation, the residual fixative was removed by washing twice with PBS. Subsequently, 0.5% crystal violet solution was added and incubated for 1 h at room temperature. Finally, the plates were rinsed with slow‐running water until the background became colorless.

### 5.21 DNA Damage Assessment (γ‐H2AX)

First, 1×10^5^ GL261 cells were seeded into confocal dishes. After 48 h, the cells were washed three times with PBS and fixed with 4% PFA at room temperature for 20 min. Following PBS washes, the cells were treated with a solution containing 5% FBS for 30 min, followed by incubation with 0.3% Triton X‐100 in PBS at room temperature for 10 min. After removing the supernatant, the cells were incubated with rabbit anti‐mouse γ‐H2AX primary antibodies (Servicebio, 1:200 dilution) at 37°C for 2 h. The cells were then washed again with PBS and incubated with goat anti‐rabbit Alexa Fluor 647‐conjugated secondary antibodies (Servicebio, 1:1000 dilution) at 37°C for 1 h. Finally, the nuclei were counterstained with DAPI. After brief PBS washes, images were acquired using a laser‐scanning confocal microscope.

### 5.22 Calreticulin (CRT) Exposure Assay

1×10^5^ GL261 cells were seeded into confocal dishes. After 48 h, the cells were washed three times with PBS and fixed with 4% paraformaldehyde (PFA) at room temperature for 20 min. After blocking with 2% goat serum (GBS) on ice for 15 min, the cells were incubated with CRT primary antibodies diluted in PBS containing 2% GBS at 4°C for 1 h, followed by washed three times with PBS and incubated with goat anti‐rabbit Alexa Fluor 647‐conjugated secondary antibodies (Servicebio, 1:1000 dilution) at 37°C for 1 h. Finally, the cells were stained with DAPI at room temperature for 5 min in the dark and washed twice with PBS. Images were acquired using a laser‐scanning confocal microscope. For flow cytometry analysis, 100 µL of CRT‐Alexa Fluor 488 primary antibody (1:100) was added to the cell pellet. The cells were gently mixed and incubated at room temperature in the dark for 1 h. Following incubation, the cells were washed twice with PBS and then resuspended in 200–300 µL of PBS. The stained cells were transferred to flow cytometry tubes for analysis to determine the positive rate.

### 5.23 Nuclear Translocation Analysis of HMGB1

1×10^5^ GL261 cells were seeded into confocal dishes. For immunofluorescence analysis, after 48 h, the cells were washed three times with PBS and fixed with 4% PFA at room temperature for 20 min, followed by permeabilization with HBSS containing 0.1% Triton X‐100 for 10 min and three washes with PBS. Subsequently, blocking was performed with 2% BSA for 1 h. The primary antibodies (1:1000) against HMGB1 were diluted in HBSS containing 2% BSA and incubated overnight at 4°C. After incubation, the samples were washed twice with HBSS, incubated with the corresponding secondary antibodies at room temperature for 2 h, and washed three times with PBS. Next, the DAPI staining solution was added, and the cells were incubated in the dark for 5 min, followed by washing with PBS. Nuclear translocation of HMGB1 was observed using confocal laser scanning microscopy. For Western blot analysis, the cells were washed three times with PBS after 48 h. Nuclear and cytoplasmic proteins were extracted following the manufacturer's instructions of a commercial kit. Protein concentrations were determined using a BCA assay and then equalized with PBS. Equal amounts of protein were separated by SDS‐PAGE and transferred onto PVDF membranes. The membranes were blocked with a protein‐free rapid blocking solution for 10 min at room temperature, followed by incubation with primary antibodies (1:10000) overnight at 4°C. After washing with TBST, the membranes were incubated with corresponding HRP‐conjugated secondary antibodies for 30 min at room temperature. Protein bands were visualized using an enhanced chemiluminescence (ECL) reagent and captured by the ChemiDoc XRS^+^ system (Bio‐Rad, USA). Protein expression levels were quantified using ImageJ software, with β‐tubulin serving for standardization.

### 5.24 Antitumor Effects of BNCT In Vivo

The orthotopic glioma model was established by intracranial injection of 1×10^5^ GL261‐Luc cells (total volume: 5 µL) into the right striatum of mice (coordinates: 2 mm lateral to the right, depth: 2.5 mm). Tumor growth was assessed using the IVIS on day 7. Tumor‐bearing mice were randomly divided into six groups: PBS, PBS+N, PLGA‐^10^BN (^10^ B 50 mg/kg), PLGA‐^10^BN+N (^10^B 50 mg/kg), M@PLGA‐^10^BN (^10^B 50 mg/kg), and M@PLGA‐^10^BN+N (^10^B 50 mg/kg). Next, 24 h after treatment, some groups were subjected to neutron irradiation at a thermal flux of 6.96×10^8^ n cm^−2^ s^−1^ for 20 min. Tumor progression was tracked every 5 days via IVIS, whereas the intracranial tumor volume was evaluated using T2‐weighted imaging (T2WI) sequences on an 11.7T MRI system. The natural survival period was recorded throughout the experiment, and blood samples were collected for biochemical analysis.

### 5.25 Combination Therapy

Successfully established tumor‐bearing mice were randomly divided into four groups: PBS, ICB, M@PLGA‐^10^BN+N (^10^B 50 mg/kg), and M@PLGA‐^10^BN+N+ICB (^10^B 50 mg/kg). 24 h after formulation administration, some groups were subjected to thermal neutron irradiation (Epithermal Neutron Flux: 6.96×10^8^ n cm^−2^ s^−1^) for 20 min. Following thermal neutron irradiation, mice in the ICB and combination therapy groups received intraperitoneal injections of PD‐1 antibody (5 mg/kg) every other day for a total of three doses. During the treatment period, tumor growth was monitored every 5 days using an IVIS, whereas the intracranial tumor volume was quantitatively analyzed via T2WI sequences on an 11.7T MRI scanner at 15 days. Survival status was monitored and recorded throughout the experiment to evaluate the differences in therapeutic efficacy among the treatment plans.

### 5.26 Immunofluorescence and Immunohistochemistry‐Based Analyses

Tumor‐bearing mice were treated under conditions identical to those used in the BNCT experiment. On day 15, euthanasia was performed, and brain tissues were collected, fixed in 4% paraformaldehyde, and paraffin‐embedded for histological analysis. After dewaxing, brain tissue sections were subjected to H&E staining and TUNEL staining, followed by observation under a laser fluorescence microscope. The detailed immunofluorescence protocol was as follows: Paraffin sections were sequentially treated with xylene (3 × 10 min), absolute ethanol (3 × 5 min), and gradient ethanol (95% ethanol 2 × 5 min, 85% ethanol 1 × 5 min, 75% ethanol 1 × 5 min), followed by thorough rinsing with deionized water (2 × 5 min). Sections were then subjected to heat‐induced antigen retrieval in 1× antigen retrieval buffer at 95°C for 20 min, cooled naturally, and washed with 1× PBS (pH 7.4) (2 × 5 min). After drying, tissue areas were outlined using an immunohistochemical pen, blocked with immunofluorescence blocking buffer for 15 min, and incubated overnight at 4°C with diluted primary antibodies. The next day, the sections were equilibrated at room temperature for 30 min, washed with 1× PBS (3 × 5 min), and incubated with PBS‐diluted secondary antibodies for 2 h at room temperature. After thoroughly washing with PBS (3 × 5 min), the sections were mounted with an anti‐fade mounting medium containing DAPI, and the coverslip edges were sealed with nail polish. Finally, the samples were analyzed using a laser‐scanning confocal microscope.

### 5.27 Western Blot Analysis

On day 15, the mice were euthanized, and tumor tissues were collected. The tumor tissues were washed 3 times with ice‐cold PBS, cut into small pieces, and placed into grinding tubes. Three 4 mm grinding beads were added, along with 20 volumes of lysis buffer supplemented with protease inhibitors, and the tissues were homogenized using a cryogenic grinder. After grinding, the samples were lysed on ice for 30 min. Following complete lysis, the samples were centrifuged at 12 000 rpm for 10 min at 4°C. The supernatant was collected, and the protein concentrations were determined by BCA assay and adjusted to the same level by PBS. All protein samples were separated by sodium dodecyl sulfate polyacrylamide gel electrophoresis (SDS‐PAGE) and then transferred onto PVDF membranes. The membranes were blocked with a protein‐free rapid blocking solution for 10 min at room temperature, followed by incubation with anti‐PD‐L1 antibody (1:700) and anti‐GAPDH antibody (1:5000) overnight at 4°C. After washing with TBST, the membranes were incubated with HRP‐conjugated goat anti‐mouse secondary antibody for 30 min at room temperature. Protein bands were visualized using ECL reagent and captured by the ChemiDoc XRS^+^ system (Bio Rad, USA). Protein expression levels were analyzed by ImageJ software, using GAPDH for standardization.

### 5.28 Flow Cytometry‐Based Analysis

Following perfusion with saline, the tumor‐bearing hemisphere was immediately excised and placed in ice‐cold saline. After thorough rinsing to remove surface blood clots, the tumor‐bearing hemisphere was transferred to an enzymatic digestion solution. The tissue samples were digested in a 37°C water bath for 30 min, followed by filtration through a cell strainer to obtain a single‐cell suspension. The enzymatic digestion tube was rinsed, and the supernatant was removed by centrifugation at 300 g for 10 min. The cell pellet was resuspended in myelin removal reagent and transferred to a 15‐mL centrifuge tube. PBS was gently layered along the tube wall, followed by centrifugation at 3,000 g and 4°C for 10 min to collect the lower cell layer. After erythrocyte lysis and cell counting, the cell concentration in each group was adjusted to ensure uniformity for subsequent flow cytometry analysis. The splenic tissues were processed as follows: tumor‐bearing mice were perfused with saline, after which the spleens were immediately excised and placed in ice‐cold saline. The splenic tissue was thoroughly homogenized using a 70 µm cell strainer whereas PBS was used to rinse the strainer and collect the cell suspension into a 50 mL centrifuge tube. After centrifugation at 500 g for 5 min, the supernatant was discarded. An appropriate volume of red blood cell (RBC) lysis buffer was added, followed by incubation at room temperature for 5 min to lyse the RBCs. The reaction was terminated by adding PBS, and the sample was centrifuged again at 500 g for 5 min. The supernatant was discarded and the splenocytes were resuspended in 2 mL PBS. The suspension was filtered through a 70‐µm cell strainer, and cell counting was performed. The cell concentration was adjusted to a uniform level across all groups for subsequent flow cytometry.

Prior to staining, the cells were blocked at room temperature for 20 min and incubated with fluorescence‐labeled primary antibodies for 30 min. For CD206 and Foxp3 detection, the cells were first treated with ice‐cold permeabilization buffer for 30 min, followed by washing with PBS (pH 7.4) before primary antibody incubation. Finally, the stained cell suspension was transferred to a 96‐well plate and analyzed using a flow cytometer (Beckman Coulter CytoFLEX S).

### 5.29 RNA Sequencing Analysis of Tumor Tissues

After treatment, tumor tissues of all the mice from PBS+N group and M@PLGA‐^10^BN+N group were collected and washed three times with cold PBS, and total RNA was extracted using TRIzol reagent (Invitrogen) according to the manufacturer's protocol. Genomic DNA was removed by DNase I digestion (TaKaRa Bio). Messenger RNA was purified from total RNA using poly T oligo‐attached magnetic beads. After fragmentation, first‐strand cDNA was synthesized using random hexamer primers, followed by second‐strand cDNA synthesis. The library was prepared by end‐repair, A‐tailing, adapter ligation, size selection, amplification, and purification. The library was checked using Qubit and real‐time PCR for quantification, and a bioanalyzer for size distribution detection. After library quality control, different libraries were pooled based on the effective concentration and targeted data amount and then subjected to Illumina sequencing. The basic principle of sequencing is “Sequencing by Synthesis”, where fluorescently labeled dNTPs, DNA polymerase, and adapter primers are added to the sequencing flow cell for amplification. As each sequencing cluster extended its complementary strand, the addition of each fluorescently labeled dNTP released a corresponding fluorescence signal. The sequencer captures these fluorescence signals and converts them into sequencing peaks using a computer software, thereby obtaining the sequence information of the target fragment. To identify DEGs (differentially expressed genes) between the two different samples, the expression level of each gene was calculated using the fragments per kilobase of exon per million mapped reads (FPKM) method. Kyoto Encyclopedia of Genes and Genomes (KEGG) pathway analysis and Gene Set Enrichment Analysis (GSEA) were used to analyze the differentially expressed genes and gene sets.

### 5.30 Statistical Analysis

All replicates were independent of each other. Mice in the treatment groups were randomized and single‐blinded. Data presented as mean ± SD. Data were analyzed by one‐way analysis of variance (one‐way ANOVA), two‐way analysis of variance (two‐way ANOVA), or two‐tailed unpaired Student's t‐test using GraphPad Prism 8.0, and differences with a p<0.05 were considered significant. The specific analytical methods are presented in each figure caption.

## Author Contributions

**Hefa Huang**: investigation. **Huihui Chai**: formal analysis, methodology, investigation, validation. **Tianwen Li**: writing – review and editing. **Ruize Zhu**: data curation, investigation. **Qisheng Tang**: investigation. **Maoyuan Sun**: data curation, methodology, investigation. **Jiawen Chen**: writing – original draft, writing – review and editing, data curation, formal analysis, investigation, methodology, project administration. **Houshi Xu**: investigation, methodology. **Shan Jiang**: investigation, methodology. **Yue Wang**: methodology, investigation. **Qinghui Li**: data curation, formal analysis. **Haoyu You**: data curation, methodology, investigation, writing – original draft, writing – review and editing, formal analysis, project administration. **Yulai Zeng**: investigation. **Peng Wang**: data curation, software. **Songlin Yan**: investigation, methodology. **Zhifeng Shi**: writing – review and editing, resources, visualization, supervision, conceptualization, funding acquisition. **Long Gu**: resources. **Zhen Li**: investigation, methodology. **Weiqiu Ping**: investigation. **Yun Guan**: investigation. **Yanlin Teng**: investigation. **Tao Sun**: conceptualization, visualization, writing – review and editing, supervision, resources. **Beining Liu**: investigation, methodology, data curation.

## Funding

National Key Research and Development Program of China (2024YFA1210201, 2023YFC2510000, 2022YFF1202804); National Natural Science Foundation of China (82373018, 82473295); Excellent project of Shanghai Municipal Health Commission (20234Z0009); Medical innovation research project of Shanghai Science and Technology Commission (23Y11906200); Dawn Program of Shanghai Education Commission (23SG07); Fudan University Medical Engineering Integration Project (IDH2310155); Non‐profit Central Research Institute Fund of Chinese Academy of Medical Sciences (2024‐JKCS‐06).

## Conflicts of Interest

All authors declare they have no competing interests. Figures were created by Peng Wang using BioRender software (https://biorender.com).

## Supporting information




**Supporting File**: advs76216‐sup‐0001‐SuppMat.docx

## Data Availability

The datasets used and/or analyzed during the current study are available from the corresponding author upon reasonable request.
