# Supplementary material for: Biomimetic Macrophage Cell Membrane‐Based Nanoparticles for Effective Treatment of Glioblastoma Through Boron Neutron Capture Therapy Combined With Immunotherapy
Source: Adv Sci (Weinh). 2026 Jun 22;13(52):e76216. doi: 10.1002/advs.76216 (PMC13336910; doi:10.1002/advs.76216)
Supplement: Supplementary file 1 [file ADVS-13-e76216-s001.docx]

Supporting Information

Biomimetic Macrophage Cell Membrane-based Nanoparticles for Effective Treatment of Glioblastoma through Boron Neutron Capture Therapy Combined with Immunotherapy

*Jiawen Chen, Haoyu You, Hefa Huang, Huihui Chai, Ruize Zhu, Yun Guan, Qisheng Tang, Tianwen Li, Shan Jiang, Houshi Xu, Peng Wang, Yue Wang, Maoyuan Sun, Beining Liu, Zhen Li, Yulai Zeng, Weiqiu Ping, Yanlin Teng, Songlin Yan, Qinghui Li, Long Gu, Tao Sun* and Zhifeng Shi**

**Supporting Figures**

Figure S1. Height profiles of eight h-^10^BN nanoparticles.

Figure S2. XPS spectra of h-^10^BN nanoparticles. A) Survey spectrum. B) B1s spectrum. C) N1s spectrum.

Figure S3. Hydrodynamic size distribution of h-^10^BN nanoparticles dispersed in deionized water.

Figure S4. Zeta potentials of h-^10^BN (0.1 mg/mL) nanoparticles and macrophage cell membrane dispersed in deionized water.

Figure S5. Average size of PLGA-^10^BN (G1) and M@PLGA-^10^BN (G2), respectively (n = 3 samples per group), during 3-day incubation with PBS (pH 7.4).

**Figure S6.** Evaluation of the lysosomal escape capability of the nanocomposite. Representative confocal images showing the intracellular distribution of M@PLGA-DiD at different time points, along with the corresponding lysosomal colocalization analysis. M@PLGA-DiD: red; Lysosome (LysoTracker): green. Scale bars = 20 μm.

Figure S7. A-D) In vitro cytotoxicity of PLGA-^10^BN (G1) and M@PLGA-^10^BN (G2) nanoparticles at various concentrations (^10^B concentration: 0, 10, 50, 100, and 200 μg/mL, respectively) against GL261 cells (data are presented as mean ± SD, n = 3).

Figure S8. A and B) The boron concentration of the brains and tissues at 12 and 48 h after i.v injection of PLGA-^10^BN (G1) and M@PLGA-^10^BN (G2) (data are presented as mean ± SD, n = 3 mice per group).

Figure S9. Survival fraction of GL261 cells in clonogenic assay in different groups (data represent mean ± SD, n = 3). Statistical significance was calculated via one-way ANOVA with Tukey’s test: ns: non-significant, *p<0.05, **p<0.01, ***p<0.001, ****p<0.0001.

Figure S10. A) Quantitative analysis of the γ-H2AX intensity in GL261 cells on the second day after neutron irradiation in different groups. B) Quantitative analysis of CRT intensity in GL261 cells on the second day after neutron irradiation in different groups. C and D) FCM analysis of CRT expression induced by different treatments in GL261 cells on the second day after neutron irradiation, with corresponding semi-quantification results. E) Representative confocal images of the expression of the nuclear colocalization of HMGB1 in GL261 cells on the second day after neutron irradiation in different groups. Scale bar = 50 μm. F) Western blot analysis of nuclear and cytoplasmic HMGB1 expression in GL261 cells on day 2 post-neutron irradiation under different treatment conditions. G and H) Semi-quantification of Western blot results for nuclear (G) and cytoplasmic (H) HMGB1, respectively. Data represent the mean ± SD (n = 3). Statistical significance was calculated via one-way ANOVA with Tukey’s test: ns: non-significant, *p<0.05, **p<0.01, ***p<0.001, ****p<0.0001

Figure S11. Representative pathological images of tumor tissues of HE and TUNEL staining in different groups. Scale bar = 50 μm.

Figure S12. A) Statistical analysis of the fluorescence intensity ratio of caspase-3 to Ki-67 (data represent the mean ± SD, n = 3). B) Relative mean fluorescence intensity (MFI) of γ-H2AX in tumor sections from different groups (data represent the mean ± SD, n = 3). Statistical significance was calculated via one-way ANOVA with Tukey’s test: ns: non-significant, *p<0.05, **p<0.01, ***p<0.001, ****p<0.0001.

**Figure S13.** In vivo safety studies were conducted on the experimental groups. A and B) On day 15 after various treatments, the levels of ALT, AST, and ALB and the serum levels of CREA were measured (data were presented as means ± SD, n = 5). C) H&E staining was performed on heart, liver, spleen, lung and kidney tissue sections from the GL261 tumor model mice after various modes of treatment.

**Figure S14.** The evaluation of the immunogenicity of M@PLGA-^10^BN. A) Schedule of the mouse experiments for immunogenicity of M@PLGA-^10^BN. B and C) Semi-quantitative analysis of serum IL-6 levels and TNF-α in mice from different treatment groups at various time points (data were presented as means ± SD, n = 3).

Figure S15. Representative confocal images of the expression of the nuclear colocalization of HMGB1 in different groups. Scale bar = 50 μm.

Figure S16. A and B) Western blot analysis of PD-L1 expression in tumor tissues from PBS+N (G2) and M@PLGA-^10^BN+N (G6) groups and the corresponding results of the semi-quantitative analysis (data presented are the mean ± SD, n = 3). Two-tailed unpaired Student’s t-test. ns: non-significant, *p<0.05, **p<0.01, ***p<0.001, ****p<0.0001.

Figure S17. FCM gating strategy for analysis of intratumoral immune cells after different treatments. A) Gating strategy for identifying mature DCs (CD45^+^CD11c^+^CD80^+^CD86^+^). B) Gating strategy for identifying CD8^+^ T (CD45^+^CD3^+^CD8^+^) and CD4^+^ T (CD45^+^CD3^+^CD4^+^) cells. C) Gating strategy for identifying CD8^+^IFN-γ^+^T (CD45^+^CD3^+^CD8^+^IFN-γ^+^) cells. D) Gating strategy for identifying M1-like GAM (CD45^+^CD11b^+^F4/80^+^CD86^+^) cells and M2-like GAM (CD45^+^CD11b^+^F4/80^+^CD206^+^) cells. E) Gating strategy for identifying MDSCs (CD45^+^CD11b^+^Gr1^+^). F) Gating strategy for identifying regulatory T cells (Tregs) (CD45^+^CD3^+^CD4^+^CD25^+^Foxp3^+^).

Figure S18. A) Flow cytometry analysis of intratumoral MDSCs. B) Flow cytometry-based analysis of intratumoral Tregs cells.

Figure S19. The mechanism of combination therapy in vivo. A and B) Flow cytometry-based analysis of DC maturation and quantitative analysis of the proportion of DC maturation in the spleens (n = 4). C and D) Flow cytometry-based analysis of CD8^+^ and CD4^+^ T cells as well as the quantitative analysis of the proportion of splenic CD8^+^ T cells (n = 3). E and F) Flow cytometry-based analysis of intratumoral CD8^+^ IFN-γ^+^ T cells and quantitative analysis of CD8^+^ IFN-γ^+^ T cells (n = 3). G and H) Flow cytometry-based analysis of M1-type and M2-type macrophages as well as quantitative analysis of the proportion of M1-type macrophages in the spleens (n = 3). I and J) Flow cytometry-based analysis of MDSCs and quantitative analysis of the proportion of MDSCs in the spleen (n = 3). K and L) Flow cytometry-based analysis of Treg cells and quantitative analysis of the proportion of Treg cells in the spleen (n = 4). M) Quantitative analysis of the proportion of intratumoral CD4^+^ T cells in the spleen (n = 3). N and O) Quantitative analysis of the proportion of intratumoral M1-type and M2-type macrophages in the spleen (n = 3). The data presented are the mean±SD. Statistical significance was calculated via one-way ANOVA with Tukey’s test: ns: non-significant, *p<0.05, **p<0.01, ***p<0.001, ****p<0.0001.

**Figure S20.** FCM gating strategy for analysis of immune cells after different treatments in the spleens. A-F) Gating strategy for identifying mature DCs (CD45^+^CD11c^+^CD80^+^CD86^+^) (A), CD8^+^ T (CD45^+^CD3^+^CD8^+^) and CD4^+^T (CD45^+^CD3^+^CD4^+^) cells (B), CD8^+^IFN-γ^+^T (CD45^+^CD3^+^CD8^+^IFN-γ^+^) cells (C), M1-like macrophage (CD45^+^CD11b^+^F4/80^+^CD86^+^) cells and M2-like macrophage (CD45^+^CD11b^+^F4/80^+^CD206^+^) cells (D), MDSCs (CD45^+^CD11b^+^Gr1^+^) (E), regulatory T cells (Tregs) (CD45^+^CD3^+^CD4^+^CD25^+^Foxp3^+^) (F).
